# Ultrabright Föster Resonance Energy Transfer Nanovesicles: The Role of Dye Diffusion

**DOI:** 10.1021/acs.chemmater.2c00384

**Published:** 2022-07-19

**Authors:** Judit Morla-Folch, Guillem Vargas-Nadal, Edgar Fuentes, Sílvia Illa-Tuset, Mariana Köber, Cristina Sissa, Silvia Pujals, Anna Painelli, Jaume Veciana, Jordi Faraudo, Kevin D. Belfield, Lorenzo Albertazzi, Nora Ventosa

**Affiliations:** † Institut de Ciència de Materials de Barcelona, ICMAB-CSIC, Campus UAB, Bellaterra, Catalonia 08193, Spain; ‡ CIBER de Bioingeniería, Biomateriales y Nanomedicina (CIBER-BBN)Instituto de Salud Carlos III. Bellaterra, 08193, Spain; § Nanoscopy for Nanomedicine Group, Institute for Bioengineering of Catalonia (IBEC) C\ Baldiri Reixac 15-21, Helix Building, Barcelona, 08028, Catalonia, Spain; ∥ Dipartimento di Scienze Chimiche, della Vita e della Sostenibilità Ambientale, Università di Parma, Parco Area delle Scienze 17/A, Parma, 43124, Italy; ⊥ Department of Chemistry and Environmental Science, College of Science and Liberal Arts, New Jersey Institute of Technology (NJIT) 323 Martin Luther King, Jr., Blvd., Newark, New Jersey 07102, United States; # Molecular Biosensing for Medical Diagnostics Group, Biomedical Engineering, Technology Eindhoven University of Technology (TUE) Eindhoven, 5612 AZ, The Netherlands

## Abstract

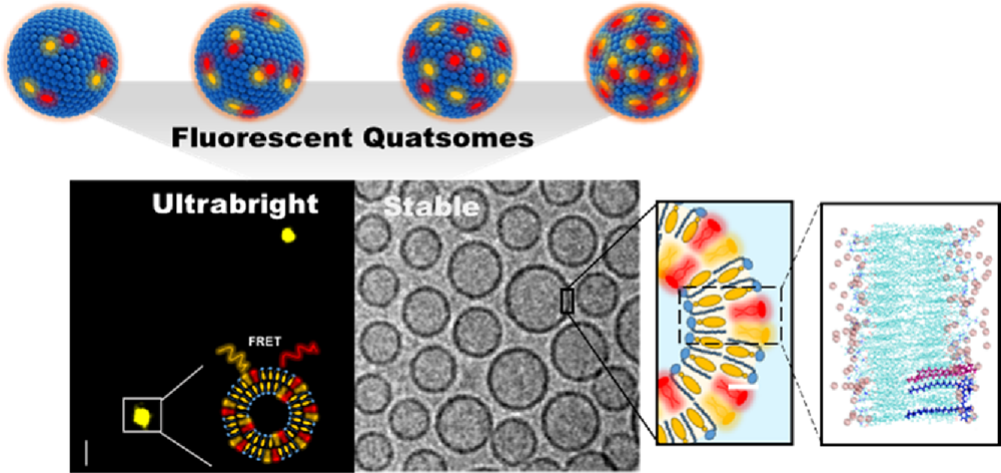

The development of contrast agents based on fluorescent nanoparticles with high brightness and stability is a key factor to improve the resolution and signal-to-noise ratio of current fluorescence imaging techniques. However, the design of bright fluorescent nanoparticles remains challenging due to fluorescence self-quenching at high concentrations. Developing bright nanoparticles showing FRET emission adds several advantages to the system, including an amplified Stokes shift, the possibility of ratiometric measurements, and of verifying the nanoparticle stability. Herein, we have developed Förster resonance energy transfer (FRET)-based nanovesicles at different dye loadings and investigated them through complementary experimental techniques, including conventional fluorescence spectroscopy and super-resolution microscopy supported by molecular dynamics calculations. We show that the optical properties can be modulated by dye loading at the nanoscopic level due to the dye’s molecular diffusion in fluid-like membranes. This work shows the first proof of a FRET pair dye’s dynamism in liquid-like membranes, resulting in optimized nanoprobes that are 120-fold brighter than QDot 605 and exhibit >80% FRET efficiency with vesicle-to-vesicle variations that are mostly below 10%.

## Introduction

Molecular imaging plays a vital role in the healthcare sector since abnormal conditions and diseases are often diagnosed through imaging, and relevant therapeutic approaches are often guided by imaging.^1^ Optical imaging is a highly sensitive technique, easier and less expensive than other imaging techniques, including tomography, magnetic resonance imaging (MRI), or ultrasound imaging.^2^ Moreover, optical imaging offers the possibility of employing multiple probes with different spectral features for multichannel imaging. The main drawback of optical imaging is the restricted tissue penetration, limited by the strong scattering and absorption of the various tissue components. Moreover, in the optical detection of single molecules, the brightness of organic dyes is limited by molar absorption coefficients lower than 300,000 M^–1^ cm^–1^ and quantum yields below unity.^3^ In this perspective, fluorescent organic nanoparticles (FONs) offer a promising alternative.

Engineering FONs exploits the flexibility offered by supramolecular synthesis and molecular self-assembly, opening the way to multifunctional probes. Moreover, FONs can harbor hundreds of fluorescent dyes to achieve enhanced brightness. FONs have proven successful in molecular imaging methods and are therefore gaining popularity in the medical imaging community.^4,5^ This spurred the recent development of several approaches to synthesize bright FONs, notably based on the direct assembly of small organic dyes into nanoparticles,^6,7^ the entrapment of fluorophores in dendrimer-like structures,^8−10^ or encapsulation of dyes in lipidic^11,12^ or polymer^13−15^ particles. However, the design of bright FONs of small diameters remains challenging mainly because the number of fluorophores per particle is limited due to fluorescence self-quenching at high dye loading, which compromises the FON brightness.^16,17^ The paradigm is even more challenging if we want to exploit Förster resonance energy transfer (FRET) at the organic nanostructure since FRET requires interchromophore distances of 1–10 nm.^18^ In FRET-based FONs, the emission signature can be fine-tuned by changing the nature, amount, and ratio of the fluorescence entities (FRET donor and acceptor). FRET FONs also allow ratiometric measurements, providing built-in self-calibration for signal correction, enabling more sensitive and reliable detection.^19^ Moreover, FRET ensures large Stokes shifts, thereby reducing background noise, and represents an attractive tool to monitor nanoparticle stability for theranostic applications. Nonetheless, there are few published works on FONs showing FRET,^20−24^ and only a few attempts were made to understand the supramolecular organization and dynamics of dyes within the nanostructure through molecular dynamics simulations (MD) or interrogating FONs with super-resolution microscopy techniques to unveil nanoscopic properties.

In previous works, we reported novel FONs built from quatsomes (QSs) loaded with lipophilic dyes.^25,26^ QSs are innovative nanovesicles made by the self-assembly of quaternary ammonium surfactants and sterols. These nanovesicles are unilamellar with a fluid-like membrane and high colloidal stability.^27^ QSs are produced through the green technology DELOS-susp, a sustainable process, which allows a high control of the molecular self-assembling process and yields nanovesicular formulations with a high degree of nanovesicle homogeneity.^28,29^ Taking into account the challenge that represents for soft materials to achieve long-term stability, QSs, which have demonstrated >3 years of colloidal stability,^30^ have aroused considerable interest in biomedical applications (i.e., as bioimaging nanoprobes).^31−34^ More recently, QSs loaded with a FRET pair composed of DiI (1,1′-dioctadecyl-3,3,3′3′-tetramethylindocarbocyanine perchlorate) and DiD (1,1′-dioctadecyl-3,3,3′,3′-tetramethylindodicarbo-cyanine perchlorate), the amphiphilic analogs of Cy3 and Cy5, respectively, were described as promising nanoprobes.^34^ Averaged spectroscopic measurements demonstrated FRET in the colloidal QS formulations, with DiI and DiD corresponding to the energy donor and acceptor, respectively.

Herein, we study and describe for the first time the impact of different dye loadings on brightness and vesicle-to-vesicle homogeneity of FRET-loaded QSs at the nanoscopic level. A set of QSs with different DiI–DiD loadings is nanoscopically investigated through direct and individual observation by stochastic optical reconstruction microscopy (STORM) and total internal reflection fluorescence (TIRF) microscopy. Characterization with super-resolution techniques in combination with conventional fluorescence spectroscopy allows the estimation of FRET efficiency and brightness at the nanoparticle level. Through this detailed inspection of the colloidal formulation, we validate the high vesicle-to-vesicle homogeneity in terms of physicochemical properties as well as spectral properties. We also show the structure and dynamics of these QS-based ultrabright stable nanoprobes with unprecedented photostability through molecular dynamics simulations reporting dye behavior at the QS fluid-like membrane. To the best of our knowledge, ultrabright nanoprobes based on photostable and biocompatible nanovesicles loaded with hundreds of carbocyanine dyes showing FRET have not been reported so far nor studied at the molecular level or explored individually at the nanoscopic level.

## Experimental Section

### Preparation of QS-I,D Formulations by DELOS-susp

3.11 mL of ethanolic solution at 7 mM of cholesterol and with different concentrations of DiI and DiD ([DiI] and [DiD]), depending on the QS-I,D formulation, was prepared (see Table S1). The mixture was kept under stirring protected from light for 20 min. The ethanolic solution containing the cholesterol and dyes was loaded into a high-pressure vessel of 7.3 mL at atmospheric pressure and the working temperature (*T*_w_ = 308 K) (Figure S2). The solution was then volumetrically expanded with compressed CO_2_ until a molar fraction (*X*_CO2_) of 0.60, reaching a working pressure (*P*_w_) of 10 MPa. The system was kept at 308 K and 10 MPa for approximately 1 h to achieve complete homogenization and to attain thermal equilibration. Afterward, the depressurization of the volumetric expanded organic phase was performed over 25.11 mL of an aqueous solution in 7 mM CTAB. Details on the DELOS-susp methodology and equipment configuration are given in Figure S2, Supporting Information. To remove the ethanol and the residual membrane components in the colloidal suspension, one step of purification was applied. In this study, we purified the as-prepared nanovesicles by diafiltration using the KrosFlo Diafiltration equipment from Spectrum Labs. In our case, a size-exclusion column of 100 kDa with a surface area of 20 cm^2^ (MicroKros, Spectrum Labs) was used. All the quatsomes were diafiltered in MilliQ-water.

### Physicochemical Characterization of Dye-Loaded QS

#### Determination of Dye Concentration in the QS-I,D Formulations and Dye Loading

To determine the DiI and DiD concentration of dye in the QS-I,D formulations, the UV–Vis absorbance of each dye was measured using a UV–vis spectrophotometer (Varian Cary 5, Agilent). QS-I,D formulations were diluted in approximately 1:10 by the addition of ethanol, in order to ensure a disruption of the QS membrane and the total solubilization of membrane components and dye molecules (keeping values of absorption unit between 0.1 and 0.3 for all final diluted samples). The concentration of each dye was determined using the Lambert–Beer law (Abs *= c ×* ε *× l*), where *c* is the concentration (M), ε is the molar extinction coefficient (M^–1^ cm^–1^), and *l* is the path length (cm), using ε^DiI^_550nm,EtOH_ = 140,000 M^–1^ cm^–1^, ε^DiD^_646nm,EtOH_ = 246,000 M^–1^ cm^–1^, and a 1 cm high-precision cell (Hellma Analytics) as a cuvette. For the determination of the loading, a known volume of each QS-I,D formulation was freeze-dried (LyoQuest-80, Telstar) at 193 K and 5 Pa for 1 week. Then, the freeze-dried dye-loaded QSs were weighed, and the loading in mass was determined using the equation (total membrane components include cholesterol, CTAB, and dyes)
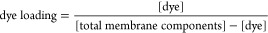


#### Nanoparticle Tracking Analysis (NTA)

The mean size and size distribution of QS-I,D 143, QS-I,D 81, QS-I,D 17, and QS-I,D 2 were analyzed by NTA using a Nanosight NS300 (Malvern Instruments) equipped with a laser at 488 nm. The laser beam passes through the sample chamber where particles in suspension scatter the light beam and can be easily visualized by a 20× magnification microscope equipped with a CMOS camera (30 fps). The video captures the particle’s movements under Brownian motion, and by using the Stokes–Einstein equation, the software determines the hydrodynamic diameter of the nanoparticles. The analysis was carried out at room temperature. Samples were diluted 10,000 times to fit the concentration range suggested by the manufacturer. The reported values are obtained as averages (*n* ≥ 3) of results from three videos for each sample.

#### Cryo-Transmission Electronic Microscopy (Cryo-TEM)

Cryo-TEM images were acquired with a JEOL JEM microscope (JEOL JEM 2011, Tokyo, Japan) operating at 200 kV under low-dose conditions. The sample was deposited onto the holey carbon grid and then was immediately vitrified by rapid immersion in liquid ethane. The vitrified sample was mounted on a cryo-transfer system (Gatan 626) and introduced into the microscope. Images were recorded on a CCD camera (Gatan Ultrascan US1000) and analyzed with the Digital Micrograph 1.8 software.

### Photophysical Properties

#### Steady-State Spectroscopy

Steady-state absorption spectra were measured with a Tecan Infinite M200 PRO plate reader spectrometer in a 1 cm path length quartz cuvette. An FLS980 fluorescence spectrometer (Edinburgh Instruments) was employed for recording fluorescence emission and excitation spectra. The fluorescence spectra were corrected for the spectral responsivity of the detector. Diluted solutions were used for fluorescence measurements, with optical densities of ∼0.1.

#### FRET Efficiency Estimate

The efficiency of energy transfer (*E*_FRET_) is the fraction of photons absorbed by the donor that are transferred to the acceptor. It can be measured using different approaches; here, the energy transfer efficiency has been determined by comparing the absorption spectrum and the excitation spectrum (through the observation of the acceptor fluorescence).^35^

#### Fluorescence Quantum Yield

The measurement of quantum yield (φ) was carried out using a Quantaurus-QY Plus (UV–NIR absolute PL quantum yield spectrometer C13534-11), Hamamatsu Photonics. The samples were diluted until absorbance values OD ≈ 0.1 were obtained. Previous to the sample measurement, a reference was measured, which consisted of the media alone. Ethanol and water were employed as reference solutions for the determination of φ of dyes in EtOH and QS-I,D, respectively. The 1 mL cuvette employed is made of synthetic quartz, which suppresses photoluminescence under UV light irradiation. The excitation wavelengths were 520 and 600 nm for DiI and DiD, respectively, illumination time was 0.9 s, and the final φ value comes from an average of 20 repetitions. These measurements were recorded at Hamamatsu Photonics GmbH (Munich, Germany).

#### Direct Stochastic Optical Reconstruction Microscopy (dSTORM)

To perform dSTORM imaging in vitro, QSs were immobilized by adsorption onto the surface of a flow chamber assembled from a glass slide and a coverslip (24 mm × 24 mm, thickness 0.15 mm) separated by double-sided tape. After being incubated for 5 min, unbound structures were removed by washing the chamber twice with STORM buffer. STORM buffer contains PBS, an oxygen scavenging system (0.5 mg/mL glucose oxidase, 40 μg/mL catalase), 5% (w/v) glucose, and 100 mM cysteamine. All images were acquired using a Nikon N-STORM system configured for total internal reflection fluorescence (TIRF) imaging. STORM images were acquired upon excitation with a 561 nm laser (nominal power, 80 mW) and after photobleaching DiD using the 647 nm laser (nominal power, 160 mW). No activation UV light was employed. 10,000 to 20,000 measurement frames were acquired using a 20 ms integration time. Fluorescence was collected employing a Nikon 100×, 1.49 NA oil immersion objective and passed through a quad-band pass dichroic filter (97335 Nikon). STORM images were analyzed with the STORM module of the NIS element Nikon software. The NIS elements Nikon software generates a list of localizations by Gaussian fitting of blinking dyes in the acquired movie of conventional microscopic images. To avoid overcounting, blinkings detected in consecutive frames are counted as single by the software.

#### Total Internal Reflection Fluorescence (TIRF) Microscopy

For TIRF-images, QSs were immobilized following the same procedure as previously described for dSTORM. 561 and 647 nm lasers were used (nominal power of 80 mW and 160 mW, respectively) in combination with a quad-band pass dichroic filter (97335 Nikon, 575–625, 660–700) for the full-emission images or a high-pass filter (670–740 nm) for the far-red images. Images were acquired onto a 256 × 256 pixel region (pixel size of 0.16 μm) of a Hamamatsu ORCA Flash 4.0 camera at 80 ms of integration time. TIRF-images were analyzed by ImageJ.

### Methods for Molecular Dynamics Simulations

#### Models and Force Fields

Cholesterol, CTAB surfactant, and water were modeled using the CHARMM36 force field as in our previous works.^27,30^ For DiI and DiD dyes, we employed the CHARMM36 compatible ForceField previously developed by us (see ref (25)) based on DFT calculations. All parameters are available at the free repository: https://bitbucket.org/icmab_soft_matter_theory/carbocianine-dyes/src/master/.

Both dyes have a total charge of +1e, distributed as shown in Figure S9. The counterion for each dye was a Cl^–^ anion.

The initial configurations of our simulations were prepared using our previous results in ref (25) as follows. From our previous work, we have an equilibrated (298 K and 1 atm) bilayer patch with a size of about 18.5 nm^2^ composed of 54 CTAB surfactants and 54 cholesterol molecules and 1 dye (DiD or DiI) in water in a box measuring 14 nm in the direction perpendicular to the bilayer. Simulations S1 and S2 with two DiI or DiD dyes were built by simply duplicating the final coordinate file from our previous work for a single dye. In the case of two different dyes, we merged the previous final files for simulations with a single DiD and a single DiD and a bilayer patch without dyes. In this way, we obtained a larger system that matches more closely typical experimental loads for FRET pairs. The resulting compositions are shown in Table S8, Supporting Information.

## Results and Discussion

**Figure 1:**
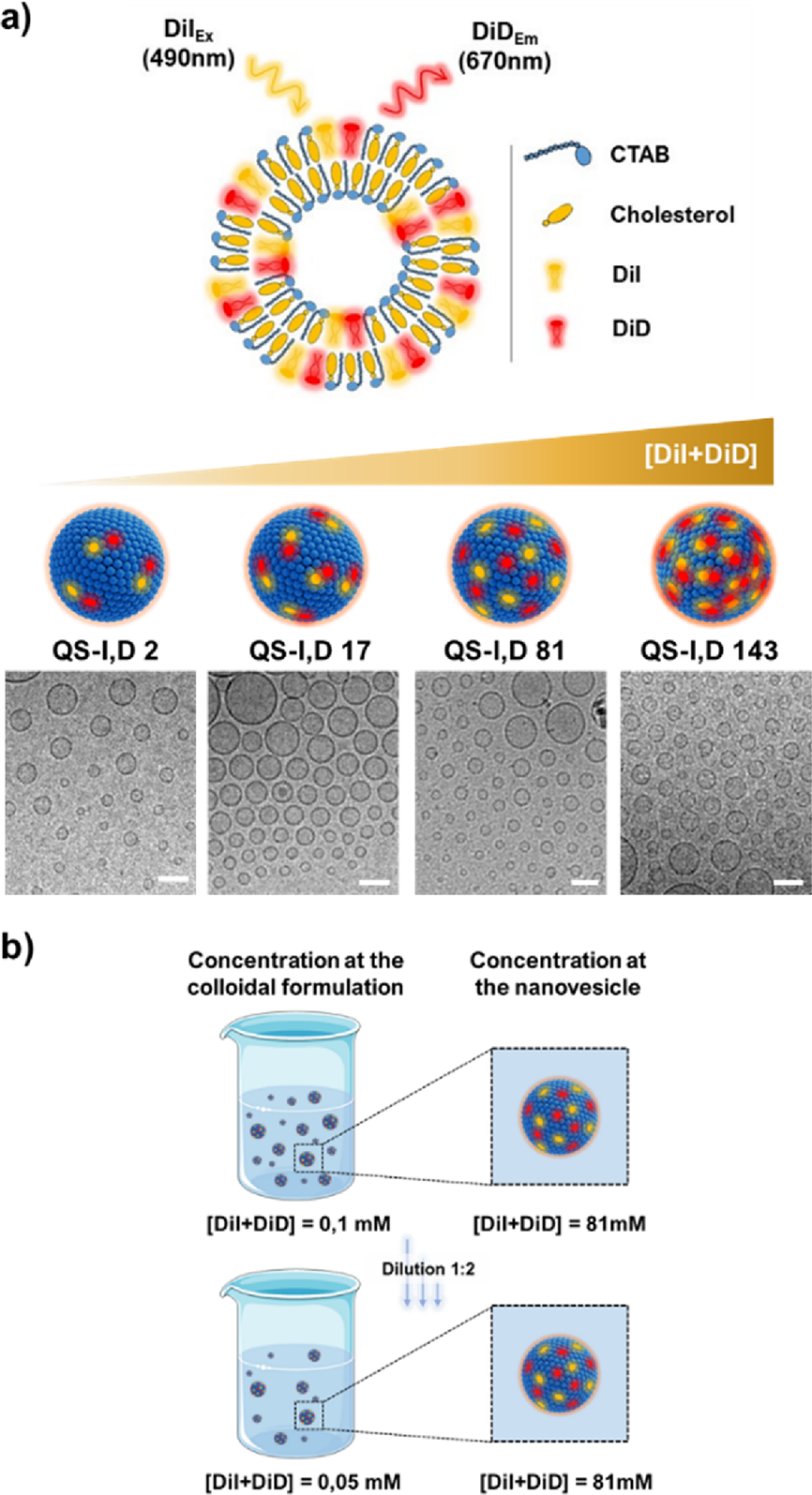
Schematic representation and characterization of the FRET QS nanovesicles under study. (a) Schematic representation of QS loaded with different amounts of DiI and DiD dyes, at an equimolar relationship between both dyes, and the representation of its components. Cryo-TEM images are displayed for each sample (scale bar = 100 nm). (b) Representation of the different impacts of dilution process over dye concentration in the bulk colloidal formulation and over dye concentration at the nanostructure.

Four different colloidal formulations of QS nanovesicles, composed of hexadecyltrimethyl-ammonium bromide surfactant (CTAB) and cholesterol, loaded with different amounts of DiI and DiD dyes but with constant equimolar relationship between the dyes (hereafter referred to as QS-I,D; Figure S1), were prepared following the DELOS-susp methodology (Figure S2 and Table S1). This method is based on the use of green compressed CO_2_ and it ensures a robust and reproducible molecular self-assembly of the nanovesicle membrane components.^25,28,29^ The final nanoformulations in aqueous media (detailed in the SI Methods section) show low nanovesicle to nanovesicle dispersity, regarding morphology and size (see Figure 1a and Table S2). In the quatsome formulation, the carbocyanine dyes are concentrated at the nanovesicle membrane compartment,^25,36^ which represents 22% of the total volume of the nanovesicle (Table S3). This means that in the final QS-I,D formulations, the dye molecules are localized in a volume that represents less than 0.3% of the total volume (Figure 1b, Table S4). This points out one of the advantages of using nanomaterials, such as QSs, to tune nanoscopic concentration without requiring an increment on the bulk concentration. To calculate the dye concentration at the nanoscale, we considered the number of fluorophores per nanovesicle and the membrane volume of the nanovesicle (Table S5). Notably, the concentration at the nanoscale is not affected by dilution since the dyes stay entrapped in the QS membrane (dilution reduces the number of nanovesicles per unit volume, but not the number of dyes in the nanovesicle, see Figure 1b). The concentration of dyes at the nanovesicle membrane provides more relevant information about the fluorophore nanoenvironment compared to the dye concentration of the bulk QS-I,D colloidal formulation, as schematized in Figure 1b. Thus, in the four QS-I,D formulations prepared, the concentrations of the membrane components (CTAB and cholesterol) were the same, while the concentration of dye at the QS nanovesicle membrane was progressively increased from 2 to 143 mM (Table 1).

**Table 1:** Nanoconcentration and FRET Brightness per Nanovesicle

	concentration per nanovesicle (mM)^a^			
sample	DiI	DiD	total	brightness_p_ (× 10^6^) (M^–1^ cm^–1^)^b^
QS-I,D 2	1	0.9	1.9	8.4 ± 0.5
QS-I,D 17	9	7.8	16.8	28.7 ± 1
QS-I,D 81	41	40	81	73.8 ± 2
QS-I,D 143	73	70	143	58.5 ± 3

a Calculated as mol dye/QS membrane volume (see Table S5), error ± 5%.

b Brightness per particle (FRET configuration) is estimated as ε_p_ × φ_F_, where φ_F_ is the fluorescence quantum yield and ε_p_ is the molar extinction coefficient at the maximum absorption wavelength of the donor specie of a single QS (Table S6).

### Physicochemical Characterization of FRET Nanoprobes at the Nanoscale

**Figure 2:**
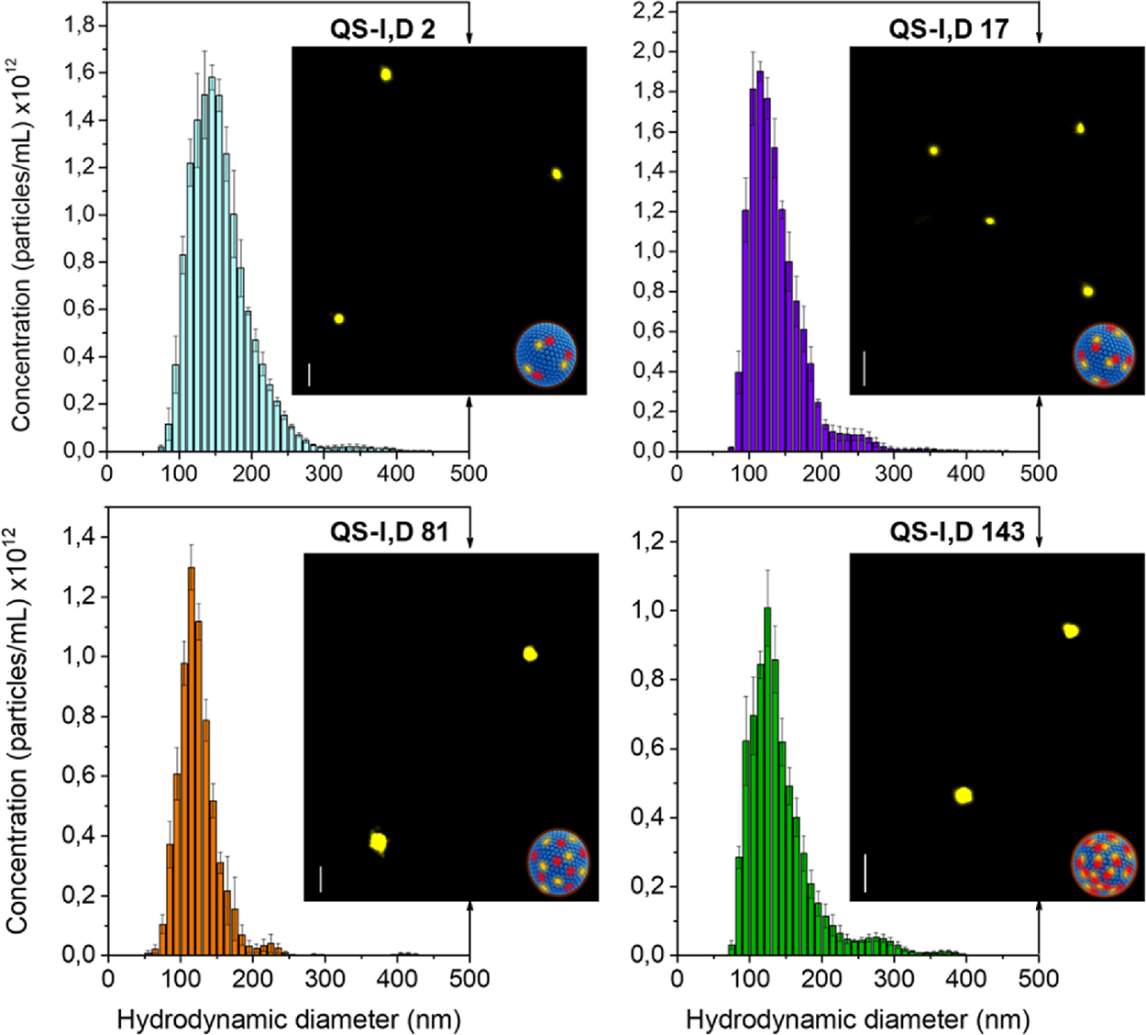
NTA and STORM of FRET QS nanovesicles. Hydrodynamic diameter distribution obtained by NTA and STORM images of four QS-I,D colloidal formulations with increasing dye concentration per nanovesicle (2, 17, 81, and 143 mM; 300 nm scale bar).

The size and morphology of the fluorescent nanovesicles were characterized by nanoparticle tracking analysis (NTA), cryo-transmission electron microscopy (cryo-TEM), and stochastic optical reconstruction microscopy (STORM). Cryo-TEM microscopy revealed spherical and unilamellar vesicles with high vesicle-to-vesicle homogeneity in terms of morphology and membrane lamellarity (Figure 1, Figure S3). NTA was employed to obtain a global size distribution after analyzing the Brownian motion of thousands of particles, yielding a mean hydrodynamic diameter of ca. 141 nm ± 11 for all four systems (Figure 2). The difference between the value reported by NTA from thousands of nanovesicles and the size distribution of *n* = 150 nanovesicles determined from different cryo-TEM (∼50 nm) could be assigned first to hydrodynamic vs geometric diameter values provided by NTA vs cryo-TEM, respectively, in addition to the lower detection limit of this latter technique. Importantly, the different dye concentration at the nanovesicles affects neither the size or morphology of the nanostructures nor the vesicle-to-vesicle homogeneity. While cryo-TEM and NTA provide information about the structural characteristics of the nanoparticles, STORM complements it with nanoscale information on fluorescence behavior.

The STORM images (Figure 2) show spherical fluorescents dots of similar sizes as the nanovesicles reported by NTA, with a mean diameter of QS-I,D of ca. 155 nm ± 9 and fairly narrow size distribution (Figure S4 and Table S2). The difference between the QS dimensions estimated via NTA and STORM can be assigned to the different resolution of the techniques and the preferential physisorption by large vs small QSs in STORM. In any case, the good agreement between NTA results (hydrodynamic diameters calculated from the nanoparticle Brownian motion) and STORM (based on the vesicle-by-vesicle inspection of fluorescence signal) confirms a very good compositional homogeneity of the nanoprobes, indicating homogeneous distribution of fluorophore molecules, independent of the loading.^28^ The controlled morphology and size of the nanoprobes at different nanoconcentrations (from 2 to 143 mM) demonstrate significant versatility of the QS nanoplatforms. Specifically, they can be loaded with different molecules^26^ in a large range of dye concentrations without compromising the size, homogeneity, or stability at the nanoscale.

### Brightness per QS Nanovesicle

The brightness of fluorescent probes constitutes an important attribute for fluorescence imaging since a particle with high brightness improves the signal-to-noise ratio, resulting in a better quality of the image and allowing for higher scanning speed.^3,37^ Brightness per particle is defined by the product of quantum yield and the extinction coefficient. Thus, in order to achieve high brightness, both the molar extinction coefficient and the fluorescence quantum yield should be maximized. Table 1 reports the estimated brightness per nanovesicle measured from spectroscopic data acquired from each nanoformulation in bulk, demonstrating that QS-I,D samples are exceptionally bright nanoparticles, with QS-I,D 81 being the best performing one. The high brightness of QS-I,D 81 (ten-fold brighter than QDots 655) turns them into ultrabright nanoparticles. As described by Sokolov and colleagues, “ultrabrightness” applies to systems whose brightness is at least one order of magnitude larger than QDs.^16,38,39^ Averaged brightness was estimated at the nanoprobes from spectroscopic data acquired from colloidal formulations, considering only the emission of the energy acceptor DiD upon exciting the energy donor to account for FRET (λ_ex_ = 520 nm). Additionally, the brightness at the nanoprobes was calculated for DiD emission when the formulation is directly excited (λ_ex_ = 600 nm), thus excluding FRET (Table S6). QS-I,D 81 displayed a brightness quantified at 7.4 × 10^7^ and 8.7 × 10^7^ M^–1^ cm^–1^ in the presence and absence of FRET, respectively. Interestingly, these data show that the brightness of QS-I,D is not compromised by FRET and points to a high FRET efficiency at the QS-nanovesicle. Table S7 displays previously reported data relevant to different fluorescent nanoparticles with emission in the NIR region, to be compared with QS-I,D with emission at λ_em_ = 650 nm. QS-I,D 81 is 120-fold brighter than QDot 605,^40^ ∼100-fold brighter than dye-loaded silica nanoparticles,^39,41^ and ∼20-fold brighter than fluorescent dendrimers.^9^ Only a few polymer nanoparticles^42^ or direct-assembled dye nanoparticles^6^ exhibit comparable brightness as QS-I,D 81. What is most relevant, most of the reported fluorescent nanoparticles are single dye loaded, while we are able to combine ultrabrightness and FRET in a single small nanoparticle characterized by very good stability. This has an enormous applicative impact as FRET-loaded QSs can be exploited for ratiometric measurements, ensure large Stokes shifts, hence improving the image quality, and allow the monitoring of the nanovesicle integrity.

### FRET Efficiency at the QS Nanovesicles

FRET efficiency is proportional to the inverse sixth power of the distance between energy donor and acceptor fluorophores and occurs typically at a distance of 1–10 nm.^18^ Therefore, we attempted to determine the optimal fluorophore loading to achieve maximal FRET efficiency without compromising the brightness. In order to evaluate the FRET efficiency in the nanovesicles, they were interrogated by conventional fluorescence spectroscopy as well as nanoscopically by TIRF microscopy. While fluorescence spectroscopy provides information about an averaged number of events occurring in the macroscopic colloidal system (where nanoparticles are in Brownian motion), the interrogation through TIRF microscopy allows a more detailed inspection of spectroscopic processes occurring in individual nanovesicles.

**Figure 3:**
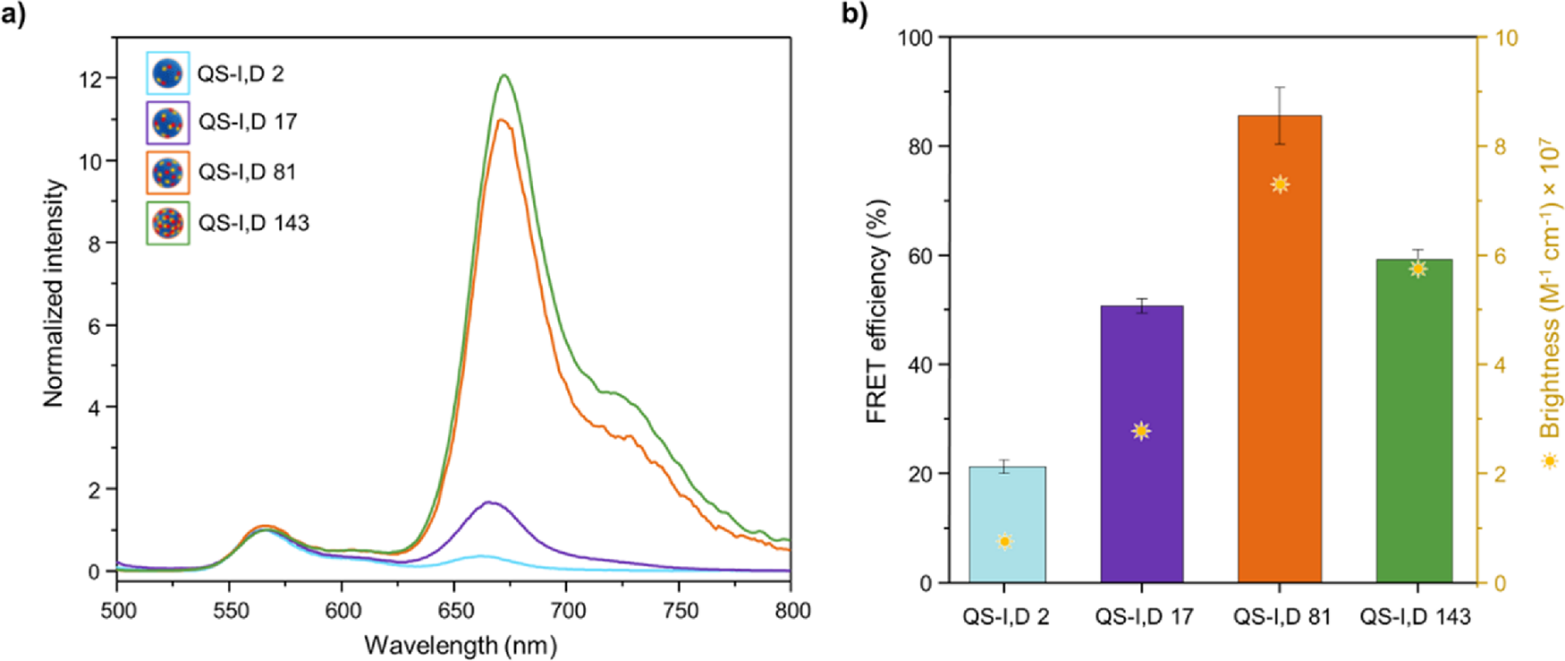
Steady-state fluorescence acquired from colloidal formulations of FRET QS nanovesicles. (a) Fluorescence emission spectra (λ_ex_ = 490 nm) normalized at donor maximum. (b) FRET efficiency calculated from the spectroscopic measurements (absorption in comparison to excitation) together with FRET brightness values reported in Table 1.

Steady-state fluorescence spectra were recorded for the nanoprobes in the colloidal formulation. Fluorescence emission spectra of QS-I,D (Figure 3a) show clear evidence of FRET occurring between DiI and DiD, upon excitation at 490 nm (where the absorption of DiD is negligible) for the four systems. Fluorescence spectra exhibit two peaks due to the DiI emission under direct excitation (λ_em_ = 570 nm) and to the FRET-induced emission of DiD (λ_em_ = 670 nm). Interestingly, the highest FRET efficiency (see the SI Methods section for the estimate of FRET efficiency) is not displayed by the formulation with the maximum loading (QS-I,D 143) but by the QS-I,D 81 nanoprobe with 85% FRET efficiency (Figure 3b). These results may indicate that, in this type of nanovesicles, dye concentrations greater than 81 mM at the QS membrane foster dye aggregation (especially for DiD, which suffers from stronger intermolecular π–π stacking),^43^ compromising the fluorescence emission performance (Figure S5). Indeed, absorption spectra revealed the formation of DiD H-dimers in nanovesicles with higher loadings (Figure S6). This hypothesis is in line with the brightness results since the brightest system is not the one with the highest fluorophore concentration at the QS nanovesicle membrane. Aggregation effects are usually observed when the local concentration is high, which can be responsible for fluorescence quenching, becoming detrimental to FRET efficiency as well as the brightness.

**Figure 4:**
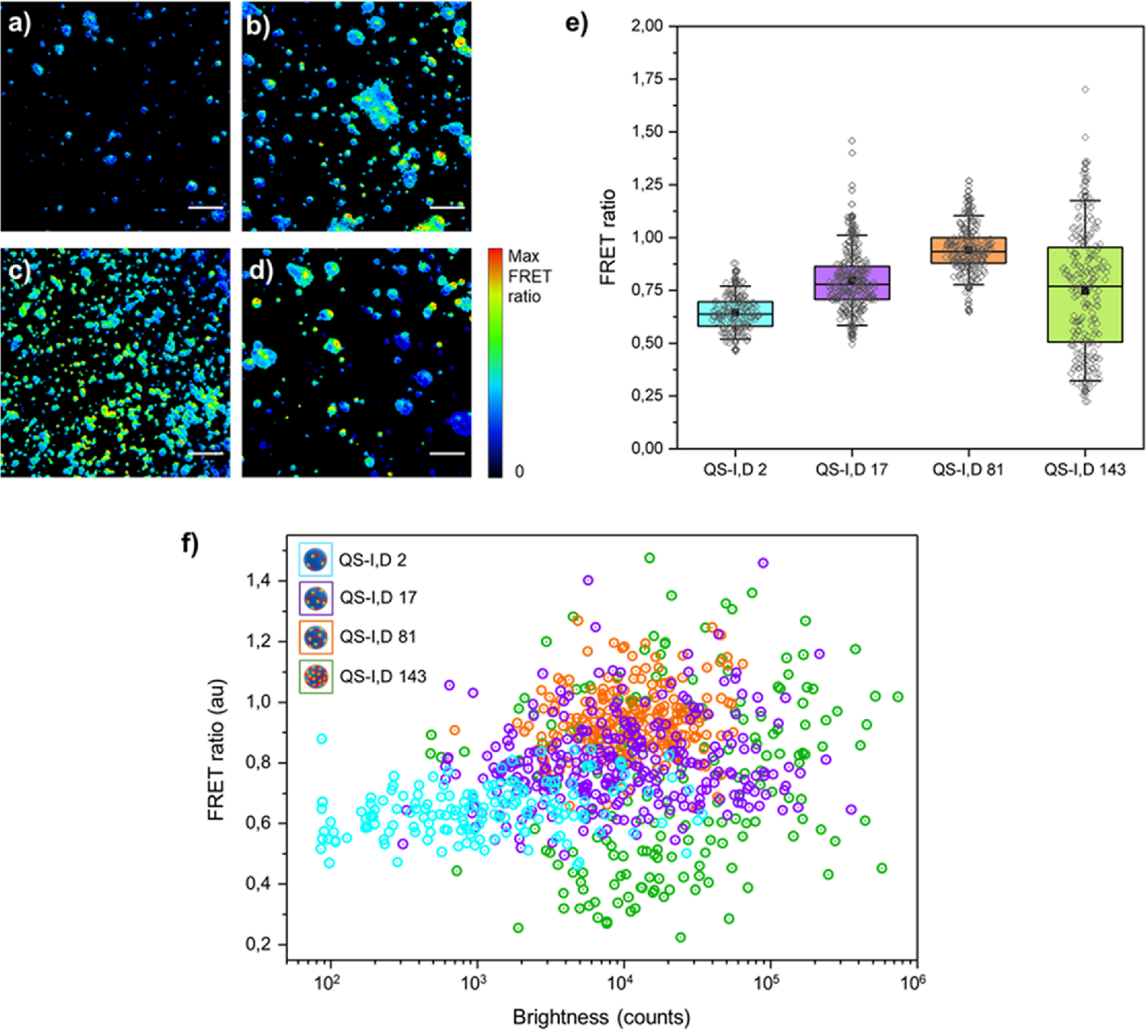
Brightness and FRET ratio studied at the single-particle level with TIRF microscopy. (a–d) FRET ratio represented from the TIRF images of QS-I,D 2, QS-I,D 17, QS-I,D 81, and QS-I,D 143, respectively (scale bar = 5 μm). (e) Box and whisker plots (indicating the 25–75 percentile and 1 SD) of the FRET ratio of individual nanoprobes, obtained from TIRF maps. Measured values are represented as empty black diamonds (*n* > 150) and mean values as filled black squares. (f) FRET ratio vs brightness of individual nanoprobes obtained by TIRF microscopy for different dye loadings.

TIRF microscopy was then exploited to assess the vesicle-to-vesicle variability of brightness and FRET ratio. Relative values of brightness and FRET ratio (as a relative measurement of FRET intensity) were obtained simultaneously from single FRET QS nanovesicles by TIRF microscopy for each QS-I,D formulation. TIRF microscopy images obtained at donor and acceptor emission were processed (Figure S7) to obtain the FRET ratio images (Figure 4a–d), used to measure the FRET ratio per particle. A box plot constructed from the FRET ratio per particle is shown in Figure 4e (Figure S8 contains the histograms). The highest mean value of the FRET ratio is displayed by QS-I,D 81. Thus, in agreement with previous spectroscopic analysis from bulk colloidal formulations, the interrogation at the nanoscopic level also points out QS-I,D 81 as the best performing nanoprobe. Importantly, QS-I,D 81 displays extremely low vesicle-to-vesicle variability of FRET ratio, where 65% of all QS-I,D 81 particles (*n* = 211) show deviations ≤10% of the mean value.

Since with TIRF microscopy, FRET ratio and brightness are obtained from single QS, Figure 4f shows a dispersion plot of these variables for each formulation. QS-I,D 2, QS-I,D 17, and QS-I,D 81 follow the same trend of increasing brightness and FRET ratio at higher dye concentration in the QS nanovesicle membrane. Importantly, among the four nanoprobes, QS-I,D 81 shows the highest vesicle-to-vesicle homogeneity of brightness being the population with the lowest variability (Figure 4f, see orange dots), also evidenced by the smaller standard deviation (Figure S9). Therefore, vesicle-to-vesicle studies as well as spectroscopic measurements in bulk confirm that QS-I,D 81 is not only the nanoparticle with high FRET efficiency and brightness but also shows a high vesicle-to-vesicle homogeneity in terms of brightness and FRET ratio, which is an important attribute for fluorescent nanoprobes. Moreover, QS-I,D 81 shows outstanding properties regarding temporal stability (up to 8 months) and also upon dilution, properties that differentiate QSs from other nanostructured systems such as micelles, which are rapidly destroyed upon dilution (Figure S10). On the other hand, QS-I,D 143 shows a significantly larger heterogeneity in comparison to the other nanoprobes. Indeed, histograms plotted from total brightness data (presented in Figure S9) as well as the larger range associated with the QS-I,D 143 box plot (Figure 4e), where 84% of all QS-I,D 143 nanoparticles show deviations >10%, clearly point to unstable photophysical behavior for QS-I,D 143 nanoparticles. We suspect that one of the major players causing this instability could be a self-quenching mechanism (as previously mentioned for this high dye loading).

At this point, it is important to emphasize the observation of FRET in formulations with low dye content (quantified at 21 and 51% FRET efficiency for QS-I,D 2 and QS-I,D 17, respectively (see Figure 3b)), as also confirmed at the single-particle level through TIRF microscopy (Figure 4e). This observation cannot be rationalized in terms of average distances. Indeed, considering a free random distribution of both dyes, the FRET pair at the QS membrane nanovesicles with low dye loadings (i.e., QS-I,D 2) has an average distance greater than the required distance for experimental FRET (Table S8). Therefore, this FRET signal observation can only be explained by considering the motion of dyes in the nanovesicle membrane. Previously, molecular dynamics simulations described the QS membrane as a liquid-like environment where DiI or DiD dye molecules experience substantial Brownian motion.^25,27^ The obtained lateral diffusion coefficients (about ∼3–4 × 10^–11^ m^2^/s at 298 K) correspond to a similar (but slightly faster) lateral diffusion to that of a hexadecyltrimethylammonium (CTA) molecular unit inside the QS bilayer or that of a typical phospholipid in a membrane. Considering these lateral diffusion coefficients^25,36^ and the surface of a QS of ∼100 nm diameter, the time required for a dye to explore the whole QS surface can be estimated by using the equation to calculate diffusion on curved surfaces defined by Faraudo.^44^ Specifically, a DiI or DiD dye molecule would need approximately 10 μs to explore the entire QS surface, making DiI-DiD encounters probable in time within the window defined by the lifetime of the excited donor.

Thus, the observation of FRET at this low nanoscopic concentration unveils important reasoning; (i) could this be interpreted as the first experimental proof of the dyes dynamism at the QS membrane and (ii) could this indicate that the encounters of donor–acceptor pairs last enough to experiment FRET?

### Molecular Dynamics Simulations

**Figure 5:**
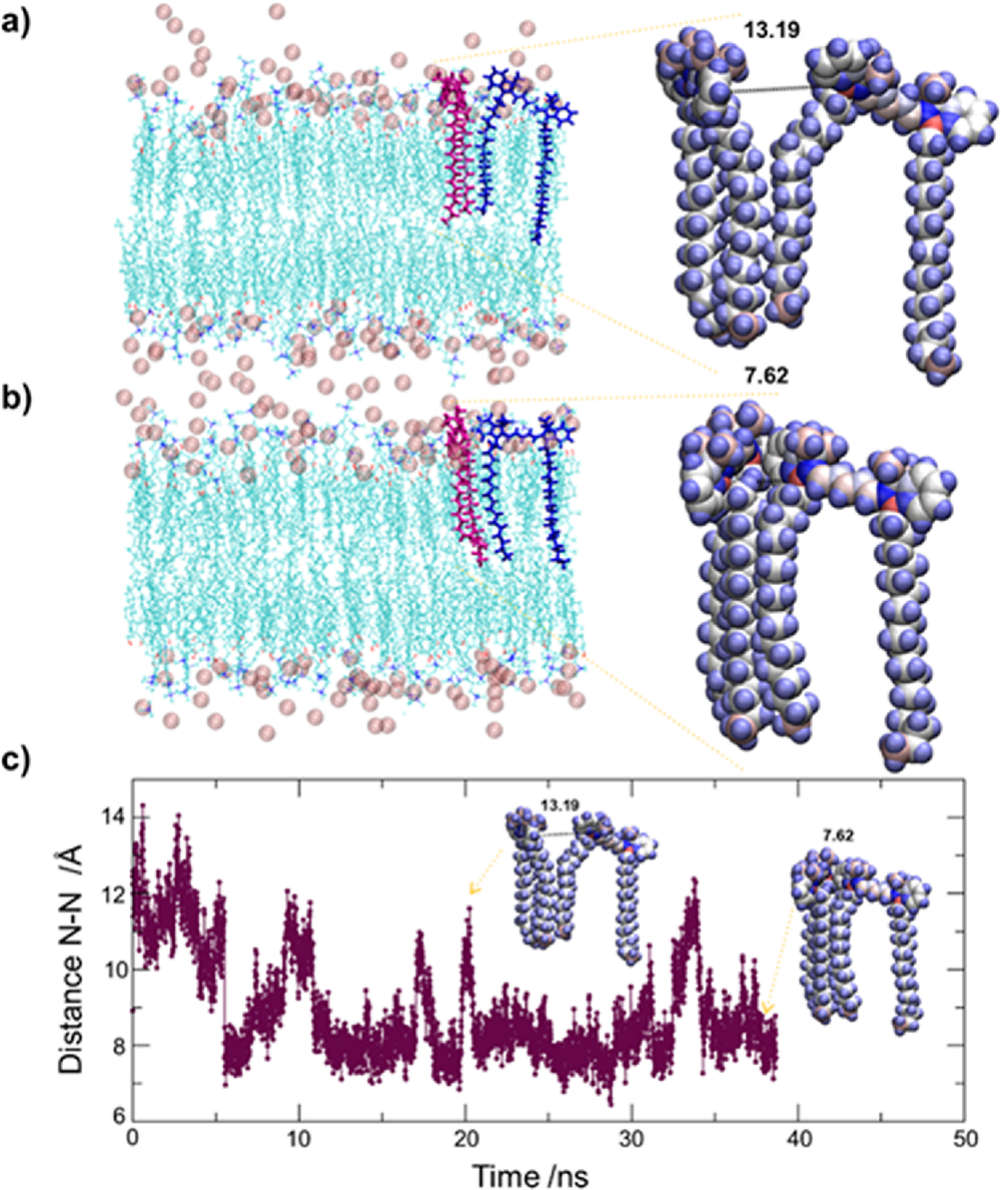
Results from MD simulations of DiI–DiD pair inside the QS bilayer. (A,B) Snapshot of a QS bilayer with the DiI and DiD molecules in a configuration with one of their hydrophobic tails in contact (a) or with their head groups in contact (b). In both cases, we show a magnification of the configuration of the two dyes. In the snapshots, all molecules are shown as lines (DiI in pink, DiD in blue, and CTA and Chol in cyan), and Br^–^ ions are shown as van der Waals spheres. Water molecules are not shown for simplicity. The enlarged molecules are shown in van der Waals representation, with atoms colored according to their partial charge (blue, positive; white, neutral; red, negative). Atomic distances are indicated in Å. (C) DiD–DiI separation (measured from their N headgroup atoms) as a function of time during the simulation with an illustration of typical configurations. All snapshots were made using VMD.^47^

We have performed further atomic MD simulations in order to study the interaction and relative motion between dyes (see the Methods section and Methods for Molecular Dynamics Simulations in the SI for details). To this end, we have considered simulations of a QS patch loaded with two identical dye molecules (Figures S11–S13) and a DiI–DiD pair (Figure 5). It is worth noting that when two dyes are inserted into a bilayer, any interaction between them is mediated by the bilayer; thus, the results should be interpreted as membrane-mediated dye–dye interaction. Our simulation of two DiI dyes inside a membrane patch indicates no significant dye–dye interaction, i.e., the two dyes diffuse as isolated dyes (Figure S12). The same simulation but with two DiD dyes (Figure S13) shows a weak DiD–DiD attraction inside the bilayer. This is consistent with our hypothesis of DiD aggregate formation (mainly H-dimers) in our experiments at high loadings (i.e., QS-I,D 143).

Finally, the simulation of a QS bilayer with a DiI and a DiD dye molecule indicates substantial interaction between DiI and DiD, as shown in Figure 5. We observed the formation of a DiI–DiD pair with two possible configurations. One configuration (Figure 5a) corresponds to interaction between one hydrocarbon chain from each dye and a clear separation between the two head groups (a N–N head group atom distance of 1.3 nm corresponding to a donor–acceptor distance of ∼1.6 nm). This configuration has typical lifetimes between ∼1 and 3 ns (see Figure 5c). The other observed dimeric configuration (Figure 5b) shows a full contact between head groups (a N–N head group distance of 0.7 nm corresponding to a donor–acceptor distance of ∼1.2 nm), with typical lifetimes of ∼5–10 ns (Figure 5c). The lifetime of these configurations lasts long enough for experimental FRET considering that the DiI lifetime at the QS membrane is ∼0.7 ns.^34^

Interestingly, two main configurations of the pair DiI–DiD are described with distances between the head groups of 0.7 and 1.3 nm (Figure 5). This observation suggests that, even if the QS membrane constitutes a liquid-like environment,^27^ DiI and DiD encounters last long enough for FRET. These results, together with the reported FRET efficiency values, point out the dynamic interplay of dyes in the QS, where DiI and DiD freely diffuse through the membrane having more donor–acceptor encounters at higher loadings. At lower loading (i.e., QS-I,D 2), the probability of such encounters is lower due to the fewer number of dyes in the membrane; as a consequence, a minor FRET efficiency is observed. Considering that the FRET process is governed by a complex interplay between different competing dynamic processes,^45,46^ additional MD simulations coupled to TD-DFT absorption/emission studies would help to understand the behavior of the FRET pair at the QS membrane. Nonetheless, these results certainly help explain the experimental observation of FRET in all QS-I,D formulations, including the ones with low dye concentration at the QS membrane.

## Conclusions

To conclude, we report the first example of FRET-based ultrabright organic nanoparticles with extremely low vesicle-to-vesicle dispersity, making them extremely interesting for fluorescence imaging. We present an extensive study where nanovesicles at different dye loadings are investigated individually at the nanoscopic level by super-resolution microscopy. This detailed inspection of the colloidal formulation allowed us to report the high vesicle-to-vesicle homogeneity in terms of physicochemical properties but also regarding optical emission, a crucial attribute for fluorescent nanoprobes. FRET efficiency as well as brightness has been estimated with TIRF microscopy and conventional fluorescence spectroscopy, reporting FRET efficiencies >80% and ultrabrightness properties. Dye-loaded QSs are interesting ultrabright nanovesicles thanks to the large local dye concentration that increases the nanovesicle extinction coefficient in an environment that avoids aggregation quenching. Ultrabright FRET-QSs however also require efficient FRET, and, in this respect, we demonstrate that a crucial role is played by the dye mobility in the QS membrane. Importantly, those optical characteristics can only be understood if the membrane is seen as a fluid-like environment, where dyes diffuse and interact over time, as suggested by molecular dynamics simulations. The optical characterization both in bulk and at the nanoscale demonstrates a good compositional homogeneity of the nanoparticles and elucidated the ideal local dye concentration in the nanovesicle for maximizing FRET without compromising brightness. To the best of our knowledge, QS-I,D 81 is the brightest FRET-based fluorescent organic nanoparticle that has been reported. We also show the structure and dynamics of these QS-based ultrabright stable nanoprobes demonstrating the DiI and DiD dynamic behavior at the QS fluid-like membrane. In this unique system, the dyes are located in the nanovesicle membrane, which represents 22% of the total volume of the nanovesicle and <0.3% of the total volume of the colloidal formulation. This tight localization represents an advantage in nanoparticle design with respect to other common organic nanoparticles where such a large local nanoconcentration is more challenging to achieve. The findings of this work not only demonstrate the potential of fluorescent QSs, and specifically QS-I,D 81, but also open the possibility to exploit QSs as nanoplatforms to study molecular interactions at the nanoscale. QS nanovesicles provide a colloidal, non-substrate supported nanomaterial where different molecules can be loaded simultaneously in a 2D liquid-like nanoenvironment.

## Supporting Information Available

Additional experimental details, materials, and methods, including experimental setup and molecular dynamics representations; graphs including additional spectroscopic data, histograms, and microscopic images (STORM and cryo-TEM); tables specifying the characteristics of the nanoparticles, calculations of brightness, and the composition of the systems considered in the MD simulations (PDF)

## ABBREVIATIONS

FRET: Förster resonance energy transfer
QS: quatsomes
FONs: fluorescent organic nanoparticles
MD: molecular dynamics
DELOS-susp: depressurization of an expanded liquid organic solution–suspension
DiI: 1,1′-dioctadecyl-3,3,3′3′-tetramethylindocarbocyanine perchlorate
DiD: 1,1′-dioctadecyl-3,3,3′,3′-tetramethylindodicarbo-cyanine perchlorate
STORM: stochastic optical reconstruction microscopy
TIRF: total internal reflection fluorescence
CTAB: cetrimonium bromide
NTA: nanoparticle tracking analysis
cryo-TEM: cryo-transmission electron microscopy
