# Supplementary material for: Ultrabright Föster Resonance Energy Transfer Nanovesicles: The Role of Dye Diffusion
Source: Chem Mater. 2022 Jul 19;34(19):8517–27. doi: 10.1021/acs.chemmater.2c00384 (PMC9558306; doi:10.1021/acs.chemmater.2c00384)
Supplement: Supplementary file 1 — cm2c00384_si_001.pdf [file cm2c00384_si_001.pdf]

SUPPORTING INFORMATION

Ultrabright Föster Resonance Energy Transfer nanovesicles: the role of the dye diffusion

Judit Morla-Folch, [a,b]# *Guillem Vargas-Nadal,* [a,b] *Edgar Fuentes,* [c] *Sílvia Illa-Tuset,* [a] *Mariana Köber,* [a,b] *Cristina Sissa,*[d] *Silvia Pujals,* [c] *Anna Painelli,* [d] *Jaume Veciana,* [a,b] *Jordi Faraudo,* [a] *Kevin D. Belfield,* [e] *Lorenzo Albertazzi,* [c,f] *Nora Ventosa* [a,b]*

[a] Institut de Ciència de Materials de Barcelona, ICMAB-CSIC
Campus UAB, Bellaterra, 08193 Catalonia, Spain
E-mail: jmorla@icmab.es; gvargas@icmab.es; silla@icmab.es; mkober@icmab.es; jfaraudo@icmab.es; vecianaj@icmab.es; ventosa@icmab.es

[b] Centro de Investigación Biomédica en Red CIBER-BBN.
Madrid, 28029, Spain.

[c] Nanoscopy for Nanomedicine Group
Institute for Bioengineering of Catalonia (IBEC)
C\ Baldiri Reixac 15-21, Helix Building, Barcelona, 08028, Catalonia, Spain.
E-mail: efuentes@ibecbarcelona.eu; spujals@ibecbarcelona.eu

[d] Dipartimento di Scienze Chimiche, della Vita e della Sostenibilità Ambientale
Università di Parma
Parco Area delle Scienze 17/A, Parma, 43124, Italy.
E-mail: cristina.sissa@unipr.it; anna.painelli@unipr.it

[e] Department of Chemistry and Environmental Science, College of Science and Liberal Arts
New Jersey Institute of Technology (NJIT)
323 Martin Luther King, Jr., Blvd., Newark, New Jersey 07102, United States.
E-mail: belfield@njit.edu

[f] Molecular Biosensing for Medical Diagnostics Group, Biomedical Engineering
Technology Eindhoven University of Technology (TUE)
Eindhoven, 5612 AZ, The Netherlands.
E-mail: L.Albertazzi@tue.nl

Table of Contents

Experimental Procedures

1. **Materials** **3**
2. **Methods for Molecular Dynamics Simulations** **3**

Figure S1. Representation of the DiI-DiD FRET pair in QS **5**
Figure S2. Equipment configuration of the small-scale reactor DELOS-susp **6**
Figure S3. Histograms obtained from cryo-TEM images of FRET nanoprobes **7**
Figure S4. STORM pictures and histogram of FRET nanoprobes **8**
Figure S4. Absorption and excitation spectra of QS-I,D at different loadings **9**
Figure S6. Normalized absorption spectra of QS-I,D at different loadings **10**
Figure S7. Procedure for FRET Ratio estimation through TIRF-microscopy **11**
Figure S8. FRET ratio results represented for each nanoprobe **12**
Figure S9. Brightness calculated from TIRF-microscopy **13**
Figure S10. Stability on time and upon dilution of QS-I,D 81 **14**
Figure S11. Geometry of DiI (left) and DiD (right) dyes in CPK representation **15**
Figure S12. Summary of results of simulation S1: two DiI molecules at QS bilayer **16**
Figure S13. Summary of results of simulation S2: two DiD molecules at QS bilayer **17**
Figure S14. Donor - acceptor separation during simulation S3 as a function of time **18**
Table S1. Process parameters of the Delos-susp methodology for QS preparation **19**
Table S2. Characteristics of the dye-loaded nanoformulations **20**
Table S3. Geometric parameters of the fluorescent QS **21**
Table S4. Volume of QS membrane in relation to the total volume **22**
Table S5. Calculation of the dye concentration at the QS membrane **23**
Table S6. Brightness per particle **24**
Table S7. Optical characteristics of fluorescent inorganic and organic nanoparticles reported in bibliography **25**
Table S8. Estimation of donor-acceptor averaged distance in the Quatsome bilayer **27**
Table S9. Composition of the systems considered in the MD simulations **28**

References **29**

Experimental Procedures

1. Materials

5-Cholesten-3β-ol (purity 95%) was purchased from Panreac (Barcelona, Spain). Hexadecyltrimethylammonium bromide (CTAB, BioUltra for molecular biology, purity ≥99.0%) was obtained from Sigma-Aldrich. 1,1'-dioctadecyl-3,3,3',3'-tetramethyl-indocarbocyanine perchlorate (DiI) and 1,1'-dioctadecyl-3,3,3',3'-tetramethyl-indodicarbocyanine perchlorate (DiD) were purchased from Life Technologies (Carlsbad, USA). MilliQ water was used for all the sample preparation (Millipore Ibérica, Madrid, Spain). Ethanol (EtOH) was from Teknocroma (Sant Cugat del Vallès, Spain). Carbon dioxide (CO_2, 99.9% purity) was purchased from Carburos Metálicos S.A. (Barcelona, Spain). All reagents and solvents purchased from commercial suppliers were used without further purification.

2. Methods for Molecular Dynamics Simulations

Protocols for MD simulation. All MD simulations were performed using the NAMD program.[5] The equations of motion were solved using a 2 fs time step. Electrostatic interactions were computed using the PME method (PME) with usual settings in NAMD (1 Å resolution, updated each 2-time steps). Lennard-Jones interactions were truncated at 1.2 nm employing a switching function starting at 1.0 nm. Periodic boundary conditions were employed in all directions. The temperature was kept constant at 298 K using a Langevin thermostat with a relaxation time of 1 ps. The pressure of 1 atm and zero surface tension were imposed using the anisotropic Nosé-Hoover-Langevin piston implemented in NAMD (oscillation period of 100 fs and decay time of 50 fs). In all cases, we performed an initial energy minimization and an equilibration run until temperature, pressure, and membrane area reach stable values (these runs were short since initial configurations were built from equilibrated ones). The production runs were 40 ns for each case.

Results for S1 and S2 simulations. For both the DiI-DiI and DiD-DiD dye cases (S1 and S2 in Table S7), both pairs are perfectly incorporated and interdigitated between QS components, as illustrated in the snapshots of Figure S10 and S11. As observed there, both dyes are located and oriented as expected, being the head groups at the water-surface interface and the tails completely immerse in the hydrophobic region of the membrane. Results from the DiI-DiI pair (S1 simulation) show that both dyes remain inside the QS membrane stable along simulation time, not deforming the membrane nor aggregating. The calculated thickness of the bilayer (measured from the peaks of the nitrogen-nitrogen atom distance from CTAB distribution) is 4.2 nm for S1 and 4.3 for S2. In our previous simulations with a single dye,[4] we obtained 4.2 nm for both dyes. It is also close to the value 4.3 nm for the bilayer thickness obtained in our

previous work in absence of dyes.[2] Note that MD simulations of DiI dyes in DPPC phospholipid bilayer[6] also predict the absence of aggregation.

We have computed the distance between both dyes present in S1 and S2 simulations as a function of time. As seen in Figure S10, the DiI-DiI distance (measured between the central carbon atoms of both dyes) presents a wide distribution, roughly uniform, indicating an absence of DiI-DiI interaction. In the case of DiD-DiD (Figure S11), we do not observe aggregation but there is a larger tendency of both dyes to be one near the other instead of being fully separated, as seen in Figure S11 (right). As shown in that figure, there is a peak at a separation of about 3 nm between the two central carbons of each dye.

Additional results for S3 simulations. The main results for the S3 simulation are reported in the main paper, Figure 5. Here, as additional material, we report the DiD-DiI separation shown in Figure 5c calculated differently. In Figure 5c, the separation between dyes is calculated as the separation between the N atoms of each dye. Here, in Figure S12 we also report the separation between DiI and DiD during the S3 simulation computed as the donor-acceptor separation. The separation is computed as the distance between each nitrogen atom of the DiI (donor) and the centre of mass of the chromophore of the DiD dye (acceptor).

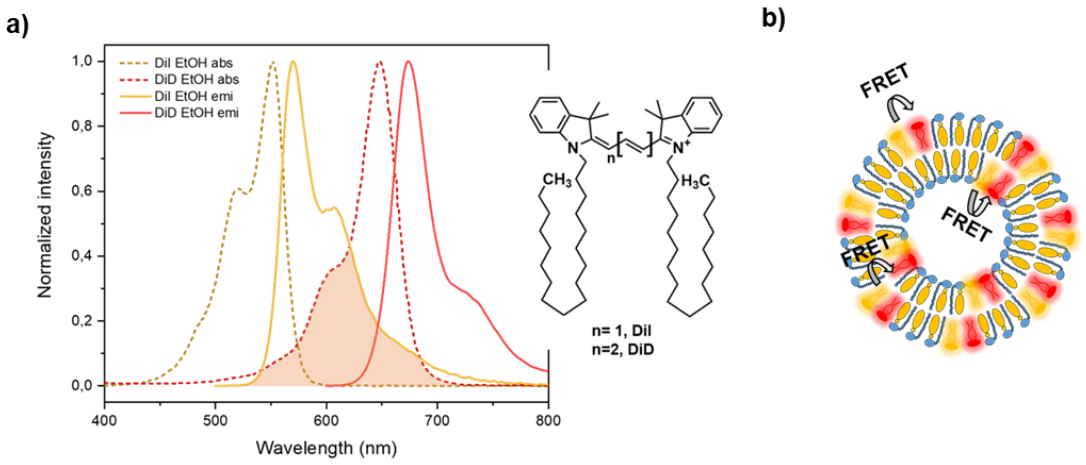

Figure S1. Representation of the DiI-DiD FRET pair in QS, named QS-I,D. a) Normalized absorption and emission spectra of DiI and DiD in ethanol. DiI and DiD are considered ideal for single-pair FRET measurements as they show well-separated absorption and emission spectra with a significant overlap (marked in orange) between the donor (DiI) emission and the acceptor (DiD) absorption. The two dyes have comparable quantum yields, and good photostability. b) Representation of a Quatsome loaded with the DiI-DiD FRET pair, called QS-I,D.

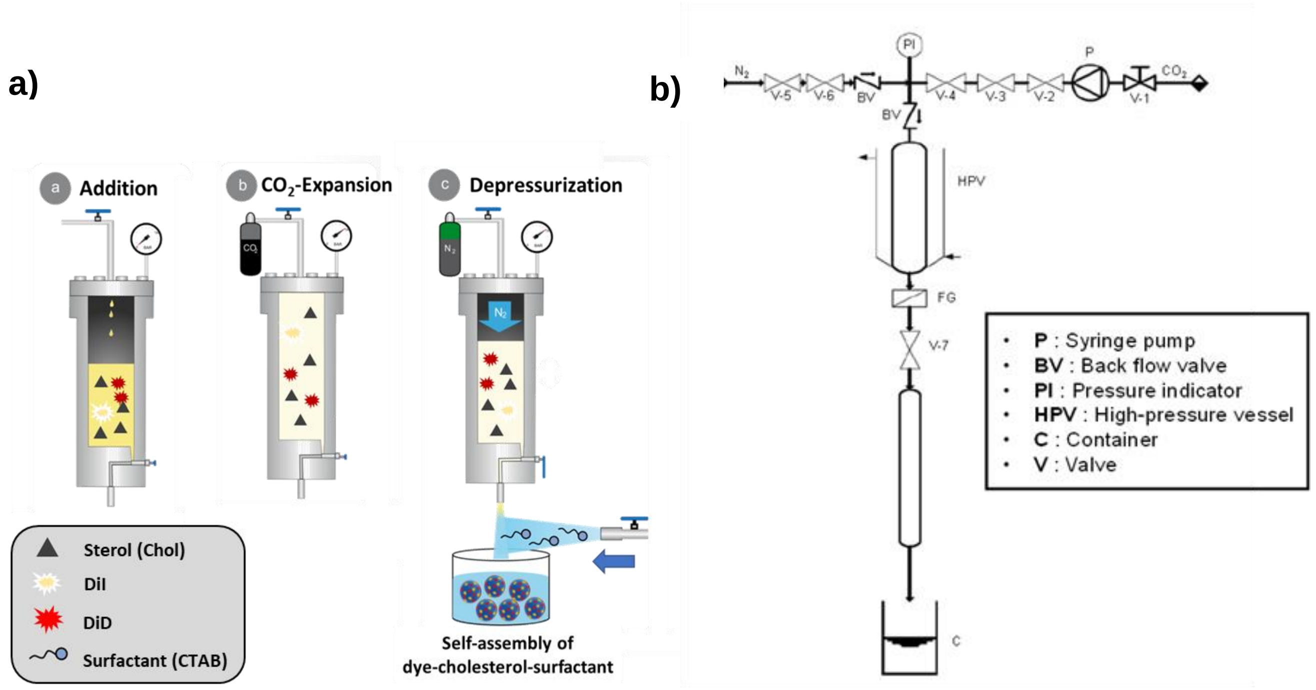

Figure S2. Equipment configuration of the small-scale reactor DELOS-susp (depressurization of an expanded liquid organic solution–suspension). Nanovesicles were prepared following the DELOS-susp procedure, schematically represented in the Figure S2 a. This method has been previously described[7] and includes the depressurization of a CO_2-expanded organic liquid solution into an aqueous phase containing a solution or dispersion of a polar compound using mild conditions of temperature (308 K) and pressure (10 MPa). a) The general procedure includes: (a) Loading of the organic solution containing the membrane components (cholesterol and dyes); (b) Addition of liquid compressed CO_2 and formation of a CO_2^- expanded solution with all the membrane components dissolved; (c) Depressurization of the CO_2^- expanded solution into an aqueous solution containing the free surfactant (CTAB). The equipment used for the preparation of QS by Delos-susp is schematized in this **Figure S2 b**. b) The configuration comprises a 7.3 mL high-pressure vessel (HPV), whose temperature is controlled by an external thermostatic bath; a syringe pump (model 260D, ISCO Inc, USA) (P) to introduce CO_2 inside the HPV through valve V-4; a depressurization valve (V-7) from which the expanded liquid solution is depressurized into the aqueous phase placed in a collector (C) located after V-7, N_2 is pumped into the vessel through V-6. A one-way valve is located after V-6 to prevent contamination of CO_2 in the N_2 line. V-2, V-3, and V-5 are dividing the CO_2 and N_2 pipelines. There is also a pressure indicator (PI) and another one-way valve before the vessel to prevent the backflow from HPV, which could lead to contamination of the gas lines.

The specific protocol for the preparation of the four QS-I,D formulations is detailed in **Table S1**.

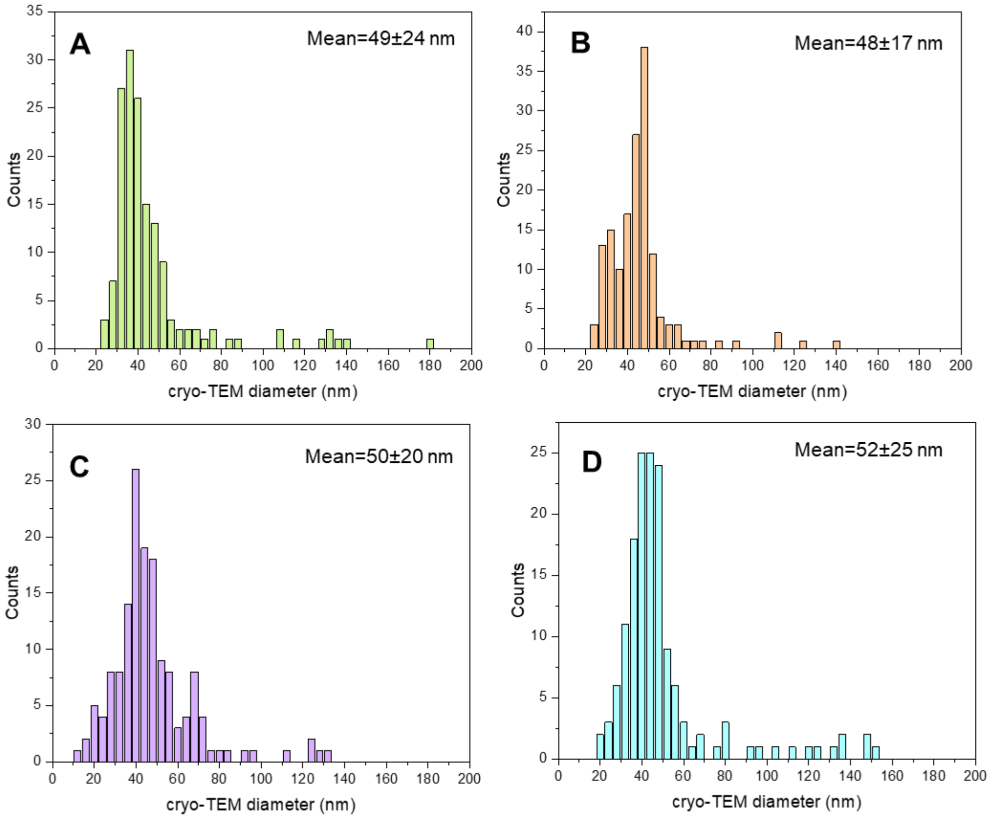

Figure S3. Histograms obtained from cryo-TEM images of FRET nanoprobes. a) QS-I,D 143; b) QS-I,D 81; c) QS-I,D 17 and d) QS-I,D 2. The histograms show the distribution of the QS diameters, measured from n=150 nanovesicles.

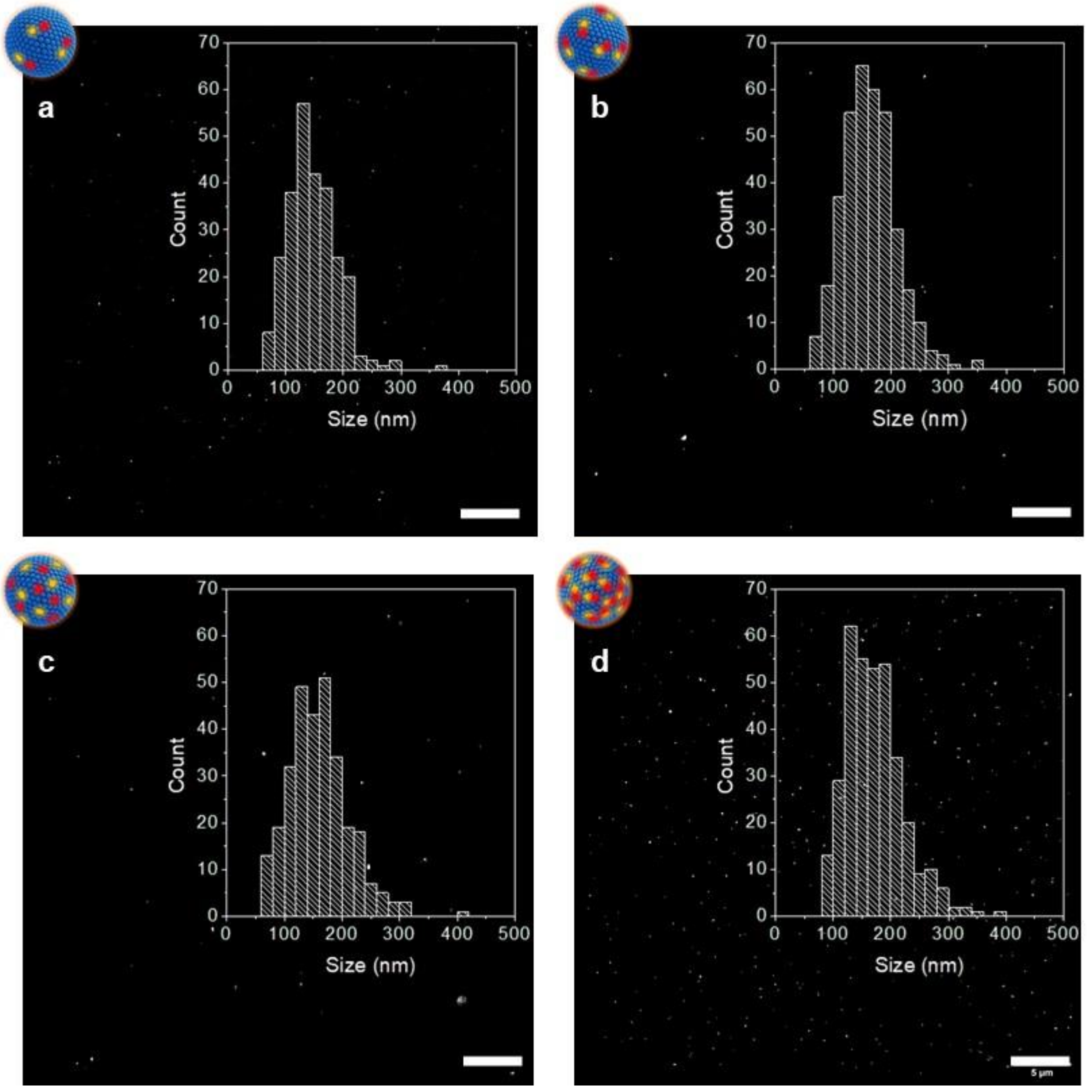

Figure S4. STORM pictures and histograms of FRET nanoprobes. STORM pictures (scale bar = 5 µm) for the QS-I,D with different loadings. a) QS-I,D 2; b) QS-I,D 17; c) QS-I,D 81 and d) QS-I,D 143. The histograms show the distribution of the QS diameters, as obtained by STORM.

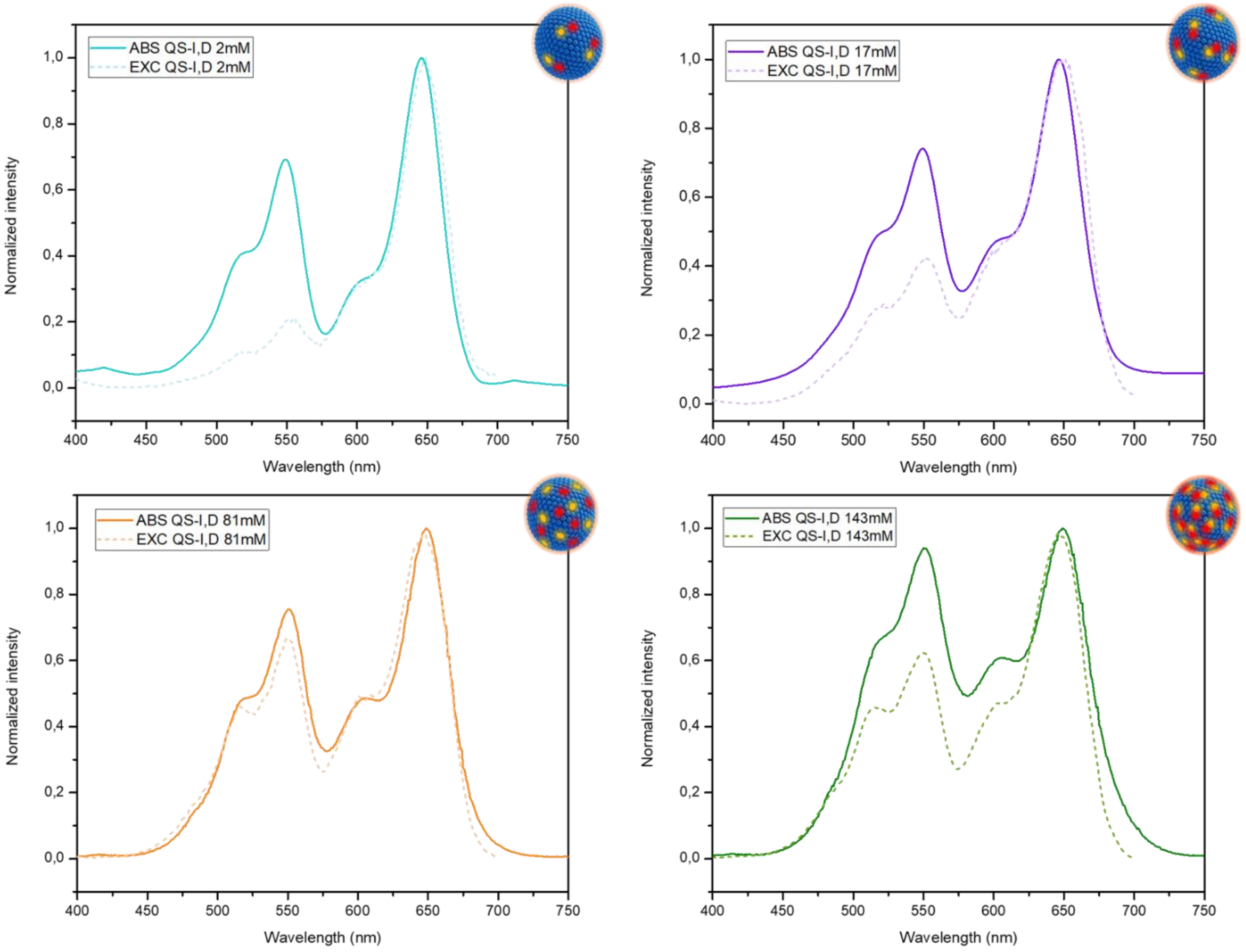

Figure S5. Absorption and excitation spectra of QS-I,D at different loadings. Diluted solutions were used for absorption and fluorescence measurements, with optical densities of ~0.1. The spectra are normalized at 650 nm. Spectra were measured in aqueous media with 1 cm path length quartz cuvette, with excitation at λ_{em} = 710 nm.

The efficiency of energy transfer is defined as the fraction of photons absorbed by the donor (DiI) which are transferred to the acceptor (DiD). It is estimated experimentally comparing the absorption spectrum with the excitation spectrum, detected from the emission of the acceptor, as in Equation 1,[1] where A_A (λ_A) and A_D (λ_D) are the maximum absorbance of donor and acceptor, at λ_A and λ_D, respectively. The fluorescence intensity of the acceptor (I_A) is indicated with two wavelengths in parentheses: the first one is the excitation wavelength, and the second is the observation wavelength.

$$\boldsymbol{\Phi_{FRET}} = \frac{A_A(\lambda_A)}{A_D(\lambda_D)}\left[\frac{I_A(\lambda_D, \lambda_A^{em})}{I_A(\lambda_A, \lambda_A^{em})} - \frac{A_A(\lambda_D)}{A_A(\lambda_A)}\right] \qquad (1)$$

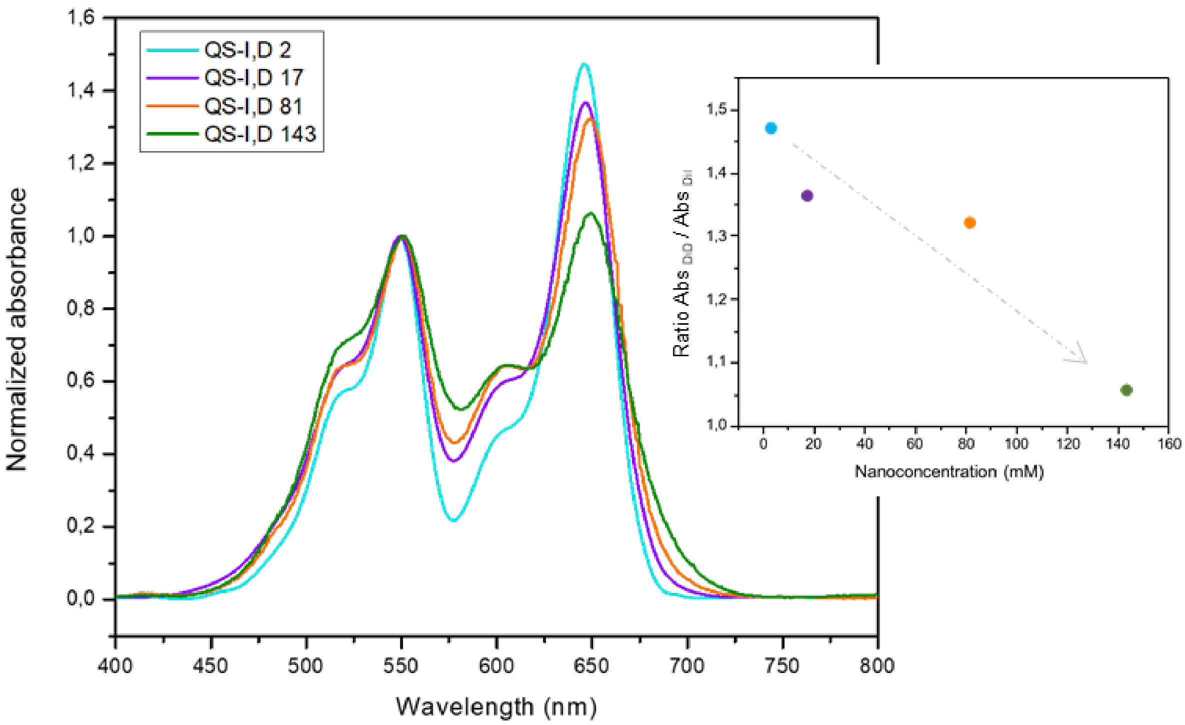

Figure S6. Normalized absorption spectra of fluorescent QS. Spectra were measured in aqueous media with 1 cm path length quartz cuvette. The spectra are normalized at DiI emission (λ_{em} = 551nm).

Absorption spectra of QS-I,D contain two peaks, the characteristic absorption bands for the monomers DiI and DiD (~552 nm and ~649 nm, respectively). The small high energy shoulder (516 nm and 600 nm, for DiI and DiD respectively) can be safely assigned as the first the vibronic replica of the monomer band,[8] attributed to the symmetric C-C valence vibration of the polymethine chain in the electronic excited state.[9] Variations in the spectral profile of absorption spectra can be ascribed to different origins, including the presence of aggregates. Several studies on cyanine dyes have investigated the relationship between the absorption bandshape and a growing population of H-dimers in solution.[10–13] As shown in **Figure S4**, when the concentration of dyes entrapped at the QS membrane is augmented (from QS-I,D 2 to QS-I,D 143) the 600 nm band becomes more prominent pointing out the presence of H-aggregates, mainly related to the absorption band of H-dimers. Moreover, although the molar ratio of two dyes was kept constant (1:1, see **Table S4**), the ratio between maximum absorbance of DiD and DiI dyes in QS-I,D changes with increasing loading. This fact is also in line with the formation of DiD H-aggregates in QS membrane at increasing loadings,[14,15] due to the hypochromism expected for H aggregates.[16]

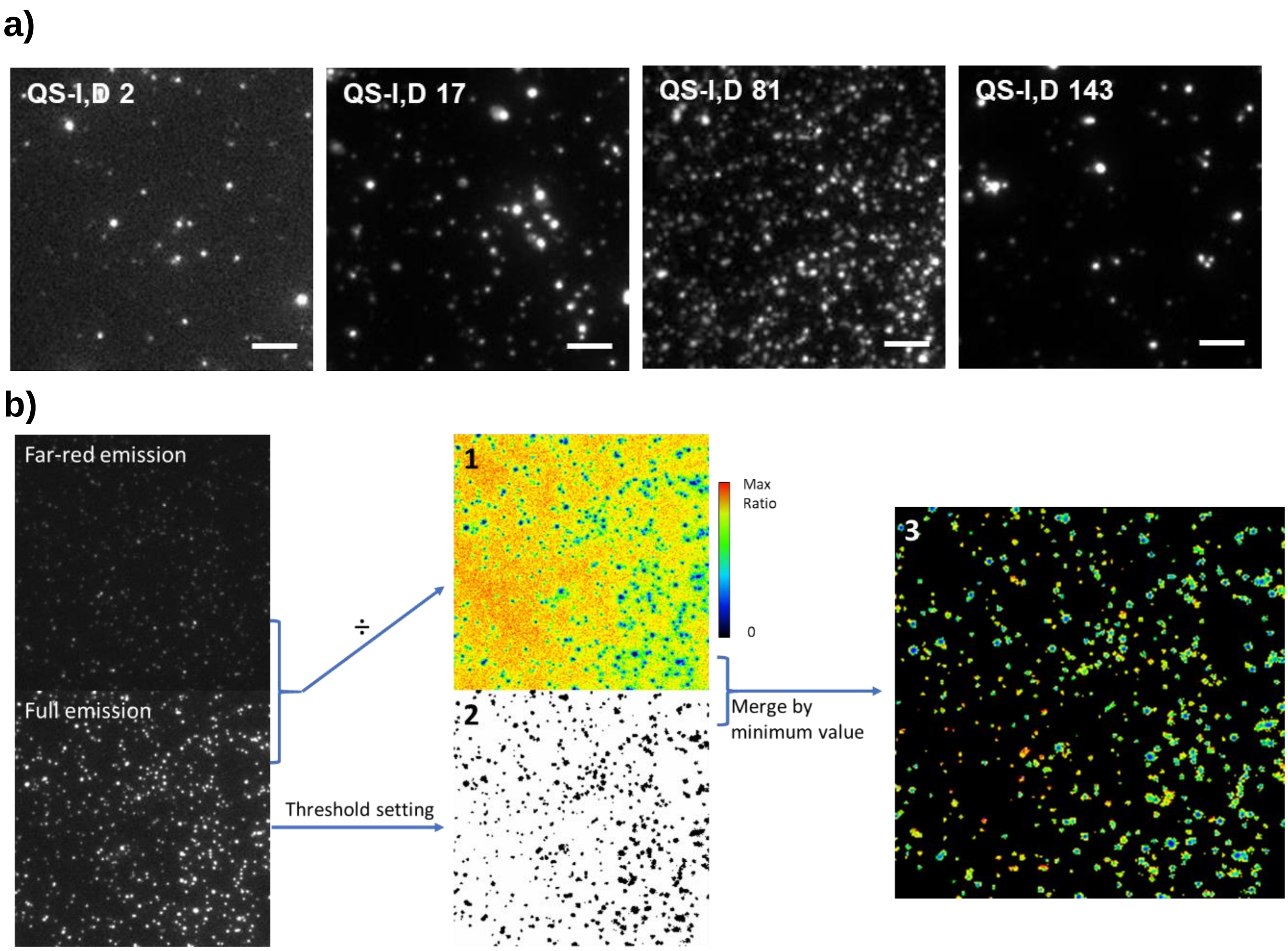

Figure S7. Procedure for FRET ratio estimation through TIRF-microscopy. a) Raw TIRF images at 561 nm of the four formulations (scale bar = 5µm). b) Schematic representation of the procedure for the calculation of FRET ratio through TIRF-microscopy.

The TIRF-images were analysed following the next steps for all the formulations:

1. Subtract background from original images. Divide the far-red emission image by the full-emission image to obtain a ratiometric image.
2. Threshold the full-emission image to obtain a mask; QS = 1, Background = NaN (Not a Number).
3. Multiply the ratiometric image by the mask. (FRET ratio information)
4. Multiply the full-emission image by the mask. (Brightness Information)
5. Stack both masked images and measure both parameters in each QS. Individual QS were easily identified in the full-emission image (output of step 4), defining the delimiting area. This same area was used to measure the brightness (output of step 4) and the FRET ratio (output of step 3).

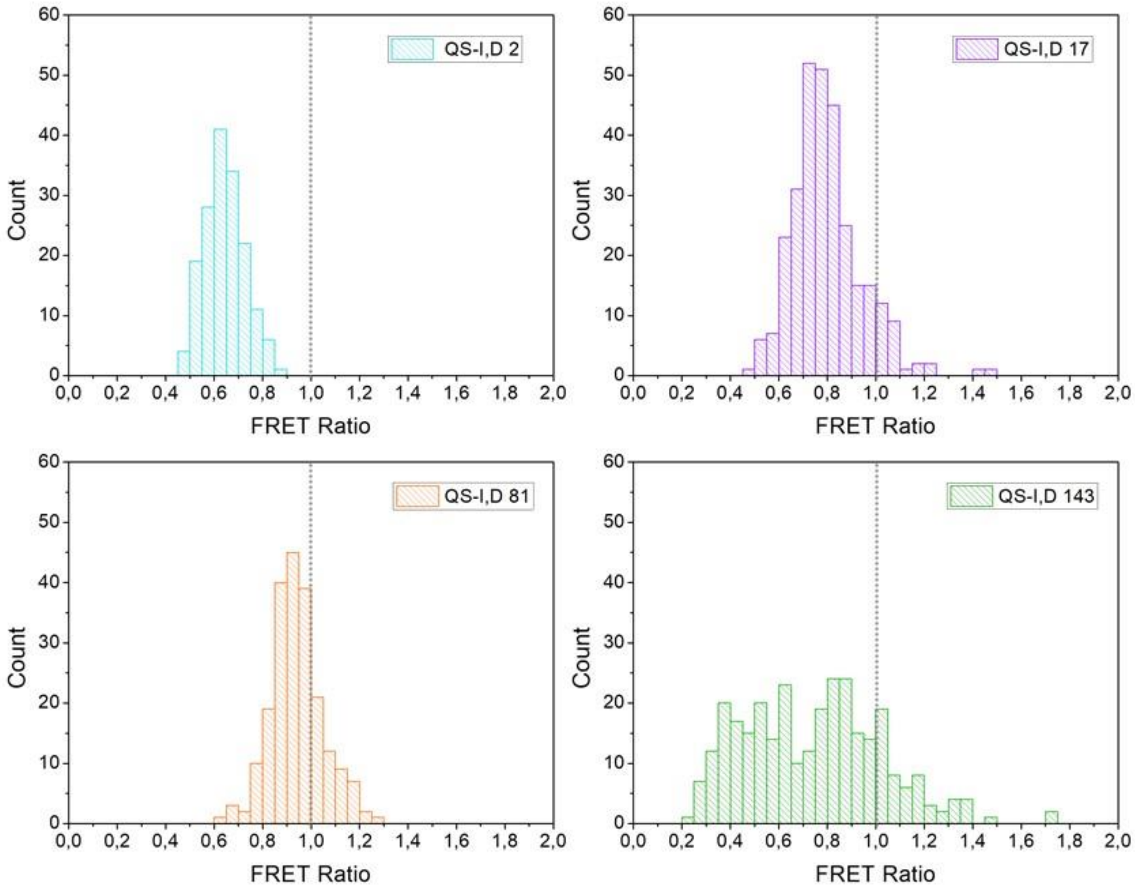

Sample	N	Mean	SD	Median
**QS-I,D 2**	166	0.645	0.083	0.638
**QS-I,D 17**	299	0.798	0.142	0.778
**QS-I,D 81**	211	0.941	0.109	0.933
**QS-I,D 143**	202	0.748	0.284	0.770

Figure S8. FRET ratio results represented for each nanoprobe (main values plotted in Figure 4e). TIRF-microscopy counts vs FRET ratio values. In the Table are presented the averaged values obtained per N nanovesicles studied (N=total number of nanoprobes interrogated, SD = Standard Deviation).

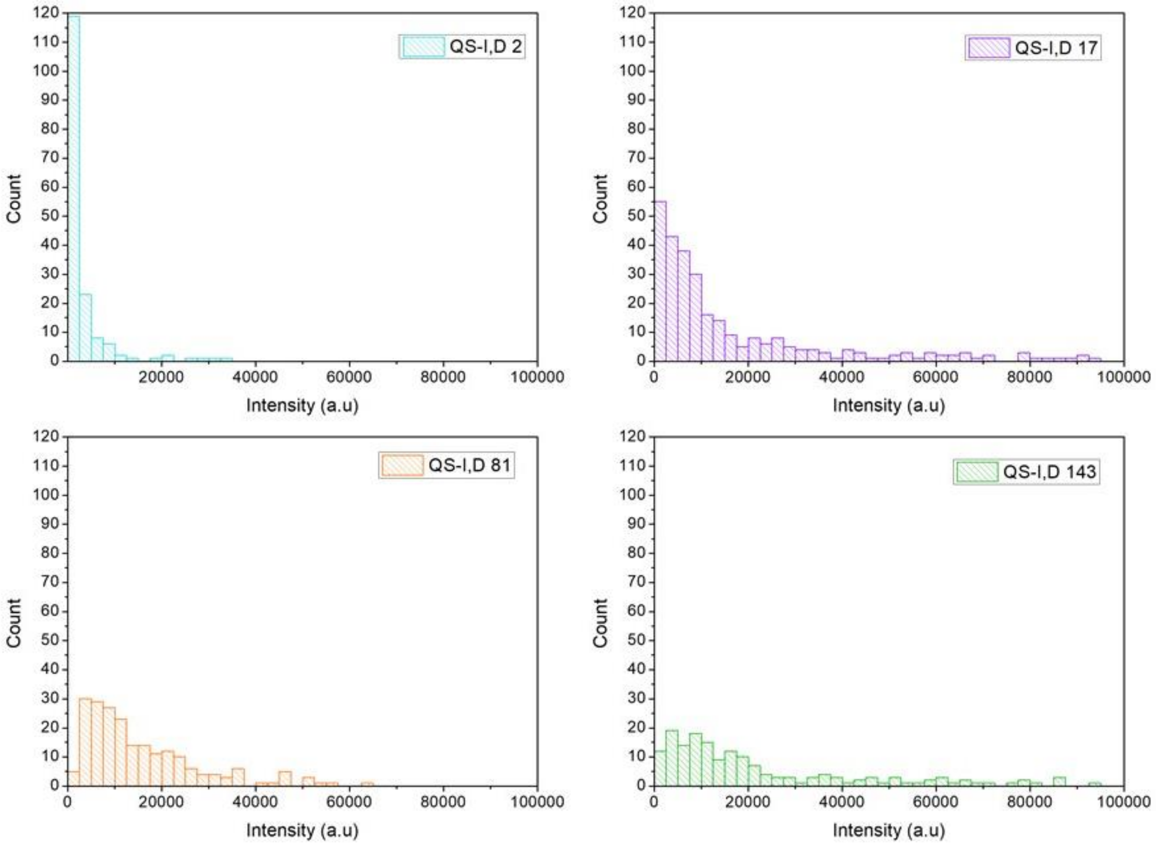

Sample	N	Mean	SD	Median
**QS-I,D 2**	166	3004.6	5556.1	1035
**QS-I,D 17**	299	22742.0	38326.5	8432
**QS-I,D 81**	211	15489.9	12360.3	11441
**QS-I,D 143**	202	59289.5	104758.1	17909

Figure S9. Brightness calculated from TIRF-microscopy. TIRF-microscopy counts vs total brightness intensity (DiI+DiD emission). In the Table are presented the averaged values obtained per N nanovesicles studied (N = total number of nanoprobes interrogated, SD = Standard Deviation).

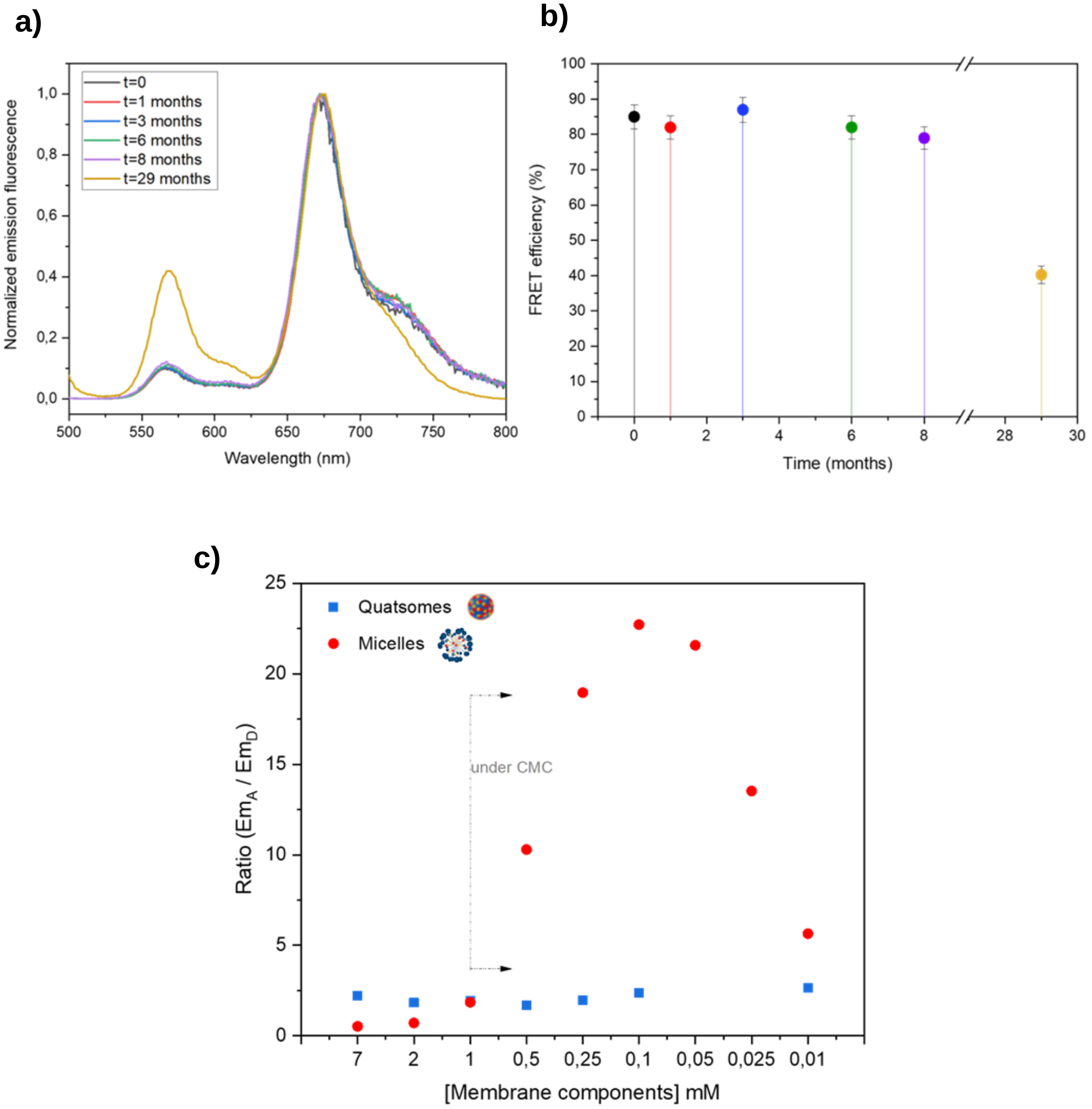

Figure S10. Stability over time and upon dilution of QS-I,D 81. (a) Fluorescence emission spectra, (b) FRET efficiency representation of the QS-I,D 81 after 1, 3, 6, 8 months and more than two years from the synthesis. (c) Micelles of CTAB loaded with DiI and DiD at same concentration as QS-I,D 81 were progressively diluted in aqueous media. Under 1 mM (the critical micellar concentration of CTAB micelles, referred as CMC), micelles become unstable indicated by the significant changes on the FRET ratio, otherwise, the FRET ratio of QS-I,D 81 is preserved at higher dilutions.

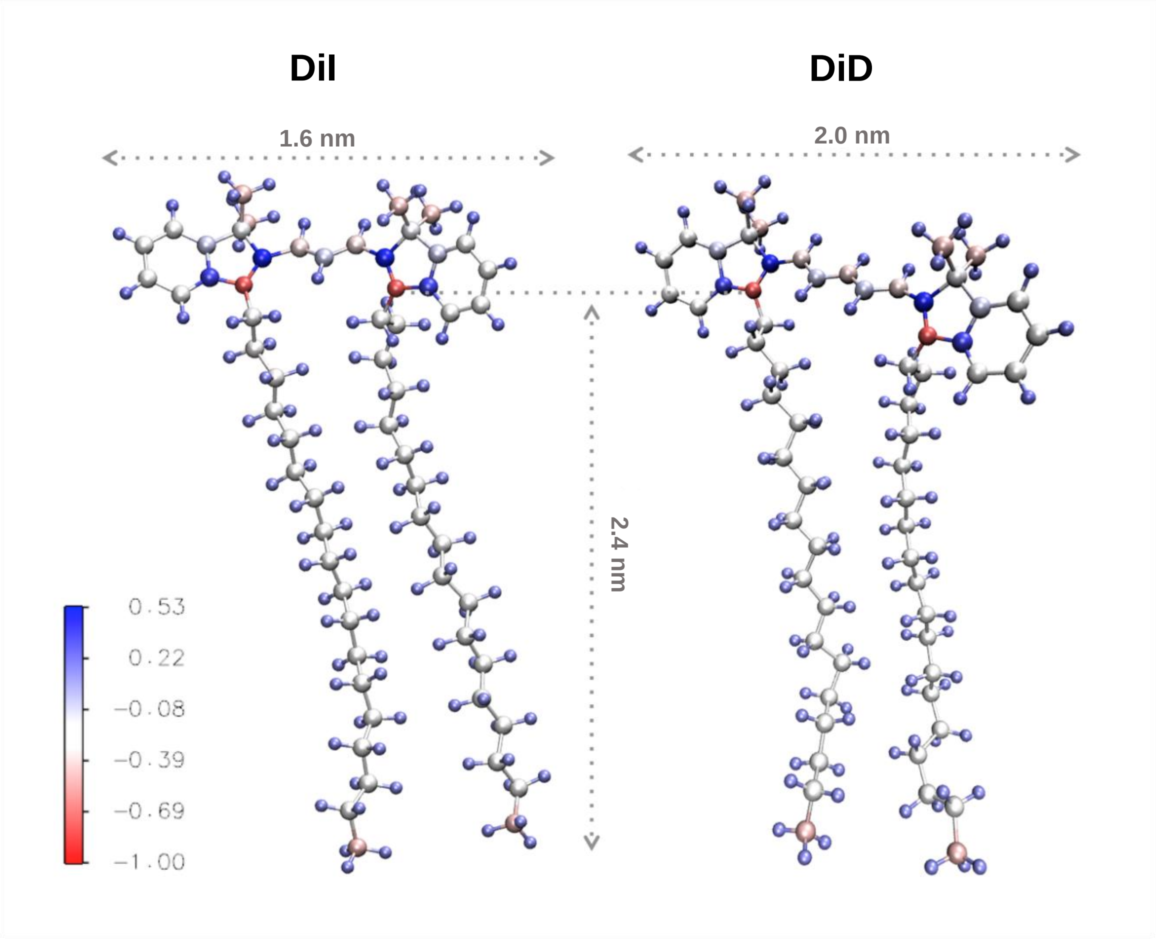

Figure S11. Geometry of DiI (left) and DiD (right) dyes in CPK representation with indication of dimensions and charge. The color scale indicates the partial charge of each atom in *e* units.

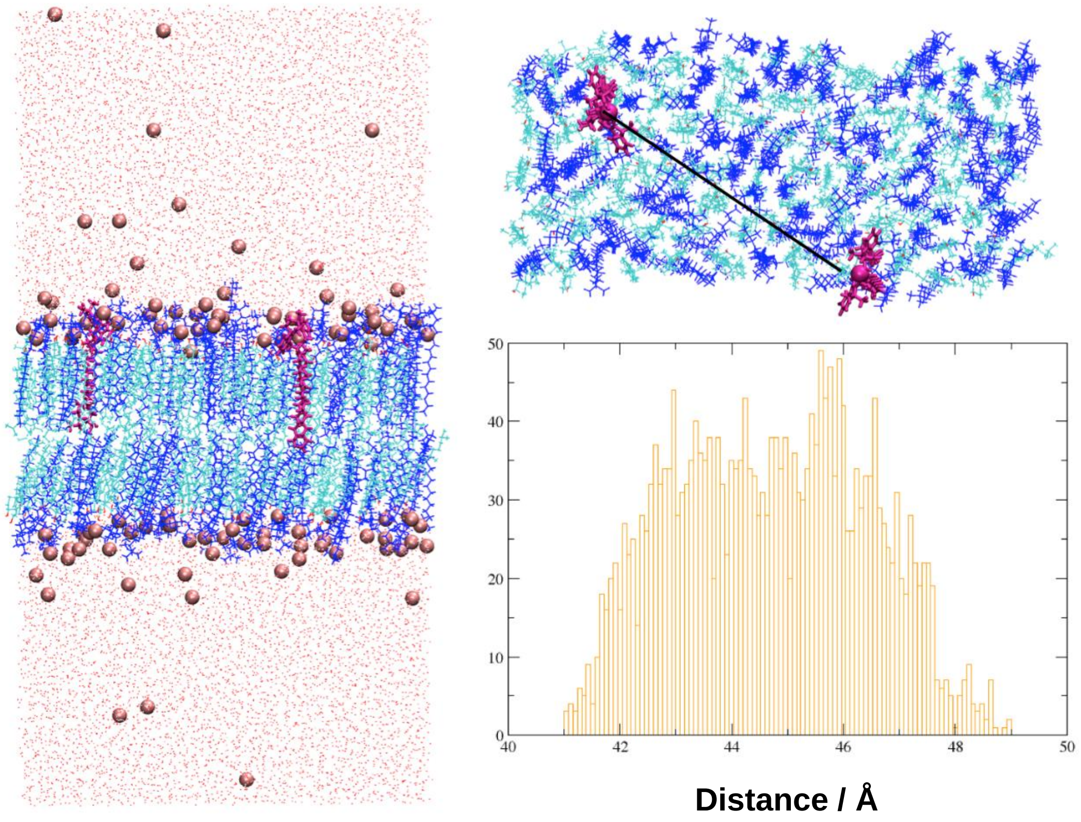

Figure S12. Summary of results of simulation S1: two DiI molecules inside a Quatsome bilayer (Table S9, Supporting Information). The snapshot of the system (front view and top view) shows both QS components CTA+ and cholesterol in blue and cyan, respectively. Water is shown as red dots and ions are shown as pink spheres. The two DiI dyes are shown in licorice representation in magenta. The histogram shows the distribution of the distance between the centre of mass of the chromophoric part of the two dyes. In the top view a black line defines the distance between the dyes.

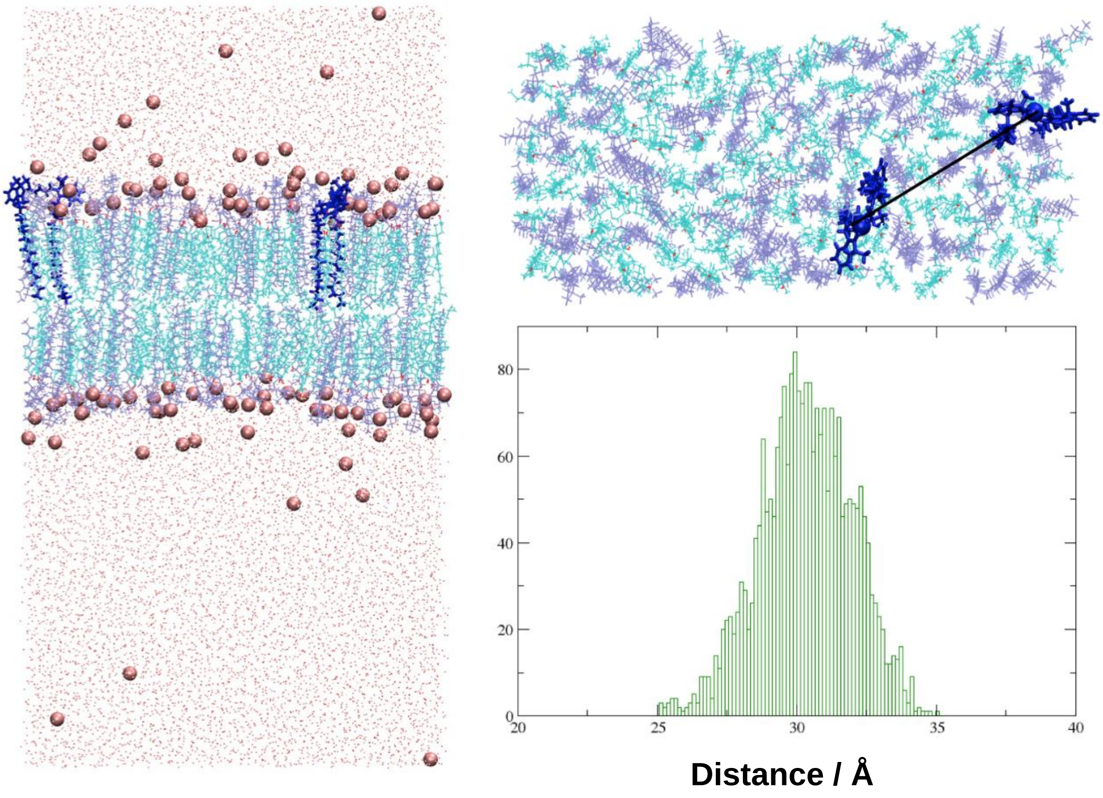

Figure S13. Summary of results of simulation S2: two DiD molecules inside a Quatsome bilayer (Table S9, Supporting Information). The snapshot of the system (front view and top view) shows both QS components CTA+ and cholesterol in grey and cyan, respectively. Water is shown as red dots and ions are shown as pink spheres. The two DiI dyes are shown in licorice representation in dark blue. The histogram shows the distribution of the distance between the centre of mass of the chromophore part of the two dyes. In the top view a black line defines the distance between the dyes.

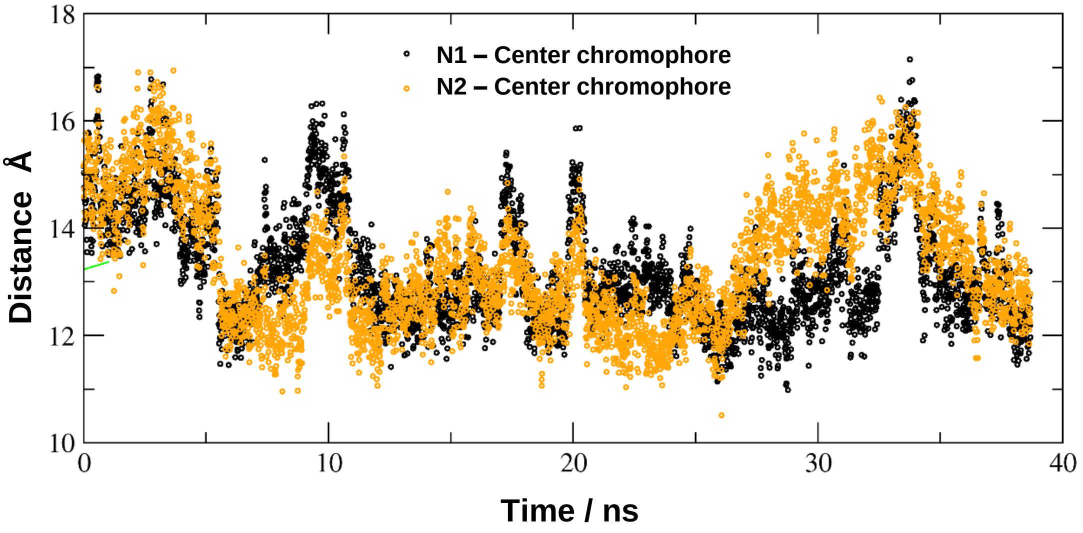

Figure S14. Donor - Acceptor separation during simulation S3 as a function of time, computed as the distance between each of the two nitrogen atoms of the DiI (donor), indicated either as N1 or N2, and the centre of mass of the chromophore of the DiD dye (acceptor).

Table S1. DiI and DiD concentrations of the ethanolic solution used for the preparation of QS-I,D formulations by Delos-susp methodology.

SAMPLE	[DiI] (mM) [a]	[DiD] (mM) [a]
**QS-I,D 2**	2.27×10^{-2}	2.27×10^{-2}
**QS-I,D 17**	2.27×10^{-1}	2.27×10^{-1}
**QS-I,D 81**	4.54×10^{-1}	4.54×10^{-1}
**QS-I,D 143**	9.07×10^{-1}	9.07×10^{-1}

[a] Dye stock solutions were used for the preparation of the initial ethanolic solution. DiI and DiD were dissolved, separately, in EtOH at a high concentration (~5 mM) and the concentration of both solutions was determined through UV-Vis spectroscopy.

For the Delos-susp experiments, 3.11 mL of ethanolic solution at 7mM of cholesterol and with different concentrations of DiI and DiD ([DiI] and [DiD]), depending on the QS-I,D formulation, were prepared (see **Table S1**). The mixture was kept under stirring protected from light for 40 minutes.The ethanolic solution containing the cholesterol and dyes was loaded into a high-pressure vessel of 7.3 mL at atmospheric pressure and the working temperature (Tw = 308 K) (**Figure S2**). The solution was then volumetrically expanded with compressed CO_2 until a molar fraction (X_{CO2}) of 0.60, reaching a working pressure (Pw) of 10 MPa. The system was kept at 308 K and 10 MPa for approximately 1 hour to achieve a complete homogenization and to attain thermal equilibration. Afterwards the depressurization of the volumetric expanded organic phase was performed over 25.11 mL of an aqueous solution at 7mM of CTAB. In this step a flow of N_2 at the working pressure is used as a plunger to push down the CO_2^- expanded solution from the vessel and to maintain a constant Pw inside the vessel during depressurization. Details of the equipment configuration are given in **Figure S2, Supporting Information.**

Table S2. Characteristics of the dye-loaded nanoformulations.

SAMPLE	[a] Dye concentration in bulk (μM)		Ratio DiI:DiD	[b] Total dye loading ($x10^{-3}$)	[c] Dye encapsulation efficiency (%)		[d] QS concentration (particle/mL) $x10^{11}$	[d] Hydrodynamic diameter (nm) *NTA*	Mean diameter (nm) *STORM*
	DiI	DiD			DiI	DiD			
**QS-I,D 2**	2.5	2.2	1.00 : 0.95	1.0	100	88	141 ± 5	144 ± 1	141 ± 44
**QS-I,D 17**	24.4	21.1	1.00 : 0.89	8.8	98	84	137 ± 9	156 ± 2	159 ± 49
**QS-I,D 81**	55	53	1.00 : 0.88	25.0	100	96	68 ± 2	138 ± 3	156 ± 58
**QS-I,D 143**	104	99	1.00 : 0.98	49.0	95	90	72 ± 4	125 ± 2	164 ± 49

[a] The final concentration of dyes was determined measuring the absorbance of the diafiltrated fluorescent Quatsomes in ethanol and applying the Lambert-Beer Law (see Experimental Section).

[b] The dye loading indicates the relation in composition between the content of dye vs membrane components. It is calculated as [(mg dye/mL solution) / (mg membrane components$_{CTAB+Chol}$ /mL solution)]. The final concentration of membrane components was estimated lyophilizing the fluorescent Quatsomes after the diafiltration (see Experimental Section).

[c] The dye encapsulation efficiency is defined as the ratio between the amount of dye present in the final formulation of QS-I,D and the initial amount of dye loaded into the reactor.

final dye concentration in bulk and the initial dye concentration in bulk.

[d] QS concentration and size distribution were measured by Nanoparticle Tracking Analysis (NTA). The averaged results are obtained from n ≥ 3 (error ± 7%)

Table S3. Geometric parameters of the fluorescent QS

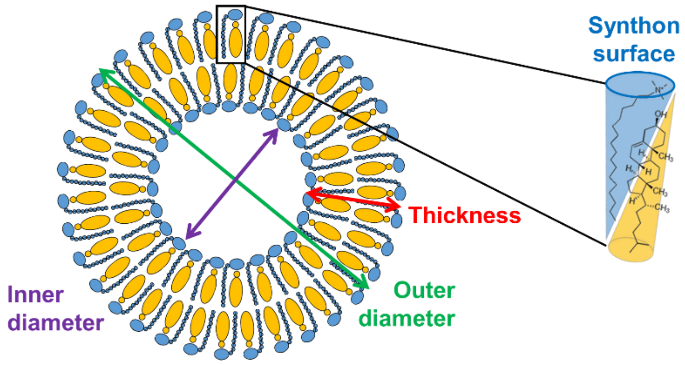

	Geometric parameters of a Chol/CTAB QS	
	[a] Diameter outer (nm)	120
	Diameter inner (nm)	110.6
	[b] Thickness membrane (nm)	4.70
VOLUME	Volume vesicle exterior (nm^3)	904778.7
	Volume vesicle interior (nm^3)	708376.3
	Membrane Volume (nm^3)	196402.4
	Membrane Volume (dm^3)	1.9 E-19
	Membrane vs nanovesicle volume	22%
SURFACE	Surface outer vesicle (nm^2)	4.52E+04
	Surface inner vesicle (nm^2)	3.84E+04
	[c] Surface synthon (nm^2)	0.57
	Number synthons at the outer QS surface	7.94E+04
	Number synthons at the inner QS surface	6.74E+04
	Number of total synthons per 1 QS	1.47E+05

[a] Average size obtained from NTA measurement and cryo-TEM pictures.

[b] Obtained from previous works, based on AFM-FS experiments [3]

[c] Obtained from Molecular Dynamics simulations [3]

Table S4. Volume of QS membrane in relation to the total volume

	Number of QS per mL	[a] Membrane volume of QS per mL	[b] QSs membrane vs total volume (%)
**QS-I,D 143**	7.24E+12	1.42E-06	0.14%
**QS-I,D 81**	6.76E+12	1.33E-06	0.13%
**QS-I,D 17**	1.37E+13	2.69E-06	0.27%
**QS-I,D 2**	1.41E+13	2.77E-06	0.28%

[a] Membrane volume of a QS obtained from Table S3 is multiplied by the number of quatsomes estimated in 1mL, determined by NTA.

[b] Membrane Relation between the QS membrane volume and the total volume in 1mL.

Table S5. Calculation of the dye concentration at the QS membrane

	QS [a]	Dye bulk concentration [b]			Dye per QS [c]			Concentration of dye per QS [d]		
	QS per mL	DiI (mol/mL)	DiD (mol/mL)	Total dye (mol/mL)	DiI per QS (mol/QS)	DiD per QS (mol/QS)	Total dye (mol/QS)	DiI at the nanodomain (M)	DiD at the nanodomain (M)	Total conc. at the nanodomain (M)
**QS-I,D 143**	7.24E+12	1.04E-07	9.90E-08	2.03E-07	1.44E-20	1.37E-20	2.80E-20	7.31E-02	6.96E-02	1.43E-01
**QS-I,D 81**	6.76E+12	5.50E-08	5.30E-08	1.08E-07	8.14E-21	7.84E-21	1.60E-20	4.14E-02	3.99E-02	8.13E-02
**QS-I,D 17**	1.37E+13	2.44E-08	2.11E-08	4.55E-08	1.78E-21	1.54E-21	3.32E-21	9.07E-03	7.84E-03	1.69E-02
**QS-I,D 2**	1.41E+13	2.50E-09	2.20E-09	4.70E-09	1.90E-22	1.67E-22	3.56E-22	9.65E-04	8.49E-04	1.81E-03

[a] Number of QS nanovesicles obtained from NTA measurements, the averaged results are obtained from n ≥ 3 per sample (error ± 7%).

[b] Obtained from UV-Vis spectra measured in bulk

[c] Obtained by: Dye (mol/mL) / QS per mL

[d] In order to obtain the concentration; dye per QS is divided by membrane volume (dm^3) obtained from **Table S3**.

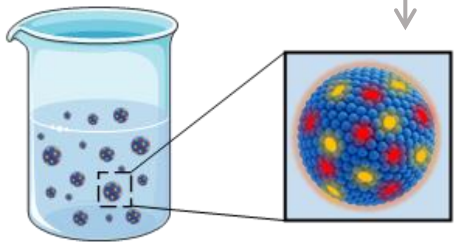

Table S6. Brightness per particle

		FRET emission [a]		DiD emission [b]	
**SAMPLE**	**ε per particle M^{-1} cm^{-1}**	**φ $_{DiD}$ (%) *Exc 520nm***	**$Brightness_p$ (x 10^6) M^{-1} cm^{-1}**	**φ $_{DiD}$ (%) *Exc 600nm***	**$Brightness_p$ (x 10^6) M^{-1} cm^{-1}**
**QS-I,D 143**	1.05E+09	5.6%	58.52	6.3%	65.84
**QS-I,D 81**	6.77E+08	10.9%	73.83	12.8%	86.70
**QS-I,D 17**	1.74E+08	16.5%	28.69	20.4%	35.47
**QS-I,D 2**	4.65E+07	18.1%	8.42	40.7%	18.94

[a] Brightness of the nanoparticle excited at DiI and emission recorded at DiD (λ_{ex} = 520 nm, λ_{em} = 670 nm)

[b] Brightness of the nanoparticle excited at DiD and emission recorded at DiD (λ_{ex} = 600 nm, λ_{em} = 670 nm)

Brightness per particle was determined as the product of the molar extinction coefficient per particle (ε_p) and the fluorescence quantum yield (φ).[17] The ε_p was determined by the Lambert-Beer Law ($\varepsilon_p = Abs_{650} / C_{QS}$ (M)), where Abs_{650} was obtained by UV-Vis spectroscopy and the molar concentration of QS (C_{QS}) from the NTA values (**Table S3**). The φ measurements were carried out using a Quantaurus-QY Plus (UV-NIR absolute PL quantum yield spectrometer C13534-11), Hamamatsu Photonics. The samples were diluted until absorbance values OD ≈ 0.1 were obtained, the excitation wavelength was 520nm and 600nm, for FRET and direct DiD emission determination, respectively, (the direct excitation of DiD at 520nm can be considered negligible).[15] Illumination time was 0.9 seconds and the final φ value come from an average of 20 repetitions.

Table S7. Optical characteristics of fluorescent inorganic and organic nanoparticles reported in bibliography.

FLUORESCENT NANOPARTICLE	SIZE (nm)	FLUOROPHORE	λ_{em} (nm)	DYE (wt %)	BRIGHTNESS (M^{-1} cm^{-1})	REF
**FLUORESCENT INORGANIC NANOPARTICLES**						
**QUANTUM DOTS**						
**QDot-585**	15-20	/	585	/	1.2×10^5	18,19
**QDot-605**	11	/	605	/	5.8×10^5	20
**QDot-655**	15	/	655	/	$\sim 10^6$	21
**DYE-LOADED SILICA NANOPARTICLES**						
**Silica core-shell NPs dye doped**	24	Rhodamine B derivative	588	0.36%	7.5×10^5	22
**Fluorophore-rich centre + siliceous shell**	20-30	TRITC	577	*[a]*	~ 2-3x QD585	23,24
**Mesoporous silica NP encapsulating dye**	28	Polymethine cyanine dye LS277	815	1.5	6.2×10^5	25
**Silica Core-Surfactant Shell NPs dye doped**	24	Rhodamine B derivative	590	~0.36%	1.5×10^5	19,26
**FLUORESCENT ORGANIC NANOPARTICLES**						
**DIRECT ASSEMBLY OF DYE NPs**						
**FONs built from Thienothiophene dyes**	27	Derivative IIIa	570	~100%	4×10^7	27
	39	Derivative IIb	711	~100%	2×10^7	
**FONs built from spirofluorene dyes**	14	JD193	716	~100%	9×10^5	28
**DENDRIMERS**						
**Phosphorous-based dendrimers (2-G_2)**	*Unknown*	Quadrupolar 2P photosensitizers	560	*[b]*	2.1×10^5	29
**Phosphorous dendrimers (G1 – G4)**	~8	TP-Chromophore	423 - 445	*[b]*	$0.75 \times 10^6 – 3.4 \times 10^6$	30

**POLYMER-BASED FNP**						
**PLGA NP**	40	Rhodamine B (R18)	590	5%	1.8×10^7	31
**PEMA-AspN3 NP**	20	Rhodamine B (R18)	590	50%	5.1×10^7	20,32
**PLGA-PEG NP**	66	DiD	665	0.5%	1.3×10^7	33
**PLGA-PEG, sample T3 (FRET cascade)**	86	DiO:DiI:DiD (1:1:0.5)	672	2.10%	$\sim 10^7$	34
**Carboxylated PSP**	100	Nile Red	635	0.74%	6.8×10^7	35
**PLGA NP**	38	Lumogen Red	605	5%	7.5×10^6	36
**LIPID NP / NANOEMULSIONS**						
**Lipid nano-droplets containing DiI**	87	DiI-TPB	553	8	7.9×10^7	37
**Fluorescent nanoemulsion (in VEA oil)**	45	Dioxaborine barbituryl styryl (DBS-C_8)	544	1.5	30×10^6	38
	50	Nile Red derivative (NR668)	563	1.5	15×10^6	
**FLUORESCENT ORGANIC NANOPARTICLES BASED ON QUASTOMES**						
**QS-D**	120	DiD	673	2.3%	1.8×10^8	15
**QS-I,D 81 (FRET)**	150	DiI:DiD (1:1)	673	4.8%	7.4×10^7	

[a] wt % not reported, however is given the concentration per particle [TRITC] = 1.43 nM/particle

[b] The photosensitisers are part of the structure of the dendrimers nanoprobes

Table S8. Estimation of donor-acceptor averaged distance in the Quatsome bilayer for the four formulations.

		QS-I,D 143	QS-I,D 81	QS-I,D 17	QS-I,D 2
DiI concentration (µM)		104,0	55,0	24,4	2,5
DiD concentration (µM)		99,0	53,0	21,1	2,2
Molecules per QS	DiI molecules	8652	4900	1073	107
	DiD molecules	8236	4722	928	94
	Total dye molec.	16888	9623	2000	201
QS surface per dye (nm^2)		5	9	42	420
[a] Theoretical average distance between two dyes in movement (nm)		2	3	7	21

[a] Dyes are constantly diffusing through the QSs membrane, at higher loadings dye molecules are closer to each other due to the higher amount of molecules per volume, leading to shorter average distances.

Table S9. Composition of the systems considered in the MD simulations.

	Atoms (total)	Num molec. DiI/DiD//water/CTA/Chol	Sim. Time	Box Size
S1 (DiI-DiI)	53892	2/0/12926/108/108	41.5 ns	32.2 nm^2 x 16 nm
S2 (DiD-DiD)	53696	0/2/12858/108/108	40.5 ns	32.1 nm^2 x 16 nm
S3 (DiI-DiD)	101248	1/1/23778/216/216	40.6 ns	91.2 nm^2 x 16 nm

Composition of the systems considered in the MD simulations, including the total number of atoms, number of molecules of each component, simulation time, and box size (for each dye molecule a Cl^- anion is also present and for each CTA^+ surfactant a Br^- counterion is also present).

References

(1) Valeur, B. B.; Nuno, M. Effects of Intermolecular Photophysical Processes on Fluorescence Emission. *Molecular Fluorescence*. 2012, pp 141–179.

(2) Ferrer-Tasies, L.; Moreno-Calvo, E.; Cano-Sarabia, M.; Aguilella-Arzo, M.; Angelova, A.; Lesieur, S.; Ricart, S.; Faraudo, J.; Ventosa, N.; Veciana, J. Quatsomes: Vesicles Formed by Self-Assembly of Sterols and Quaternary Ammonium Surfactants. *Langmuir* **2013**, *29* (22), 6519–6528.

(3) Gumí-Audenis, B.; Illa-Tuset, S.; Grimaldi, N.; Pasquina-Lemonche, L.; Ferrer-Tasies, L.; Sanz, F.; Veciana, J.; Ratera, I.; Faraudo, J.; Ventosa, N.; et al. Insights into the Structure and Nanomechanics of a Quatsome Membrane by Force Spectroscopy Measurements and Molecular Simulations. *Nanoscale* **2018**, *10* (48), 23001–23011.

(4) Ardizzone, A.; Kurhuzenkau, S.; Illa-Tuset, S.; Faraudo, J.; Bondar, M.; Hagan, D.; Van Stryland, E. W.; Painelli, A.; Sissa, C.; Feiner, N.; et al. Nanostructuring Lipophilic Dyes in Water Using Stable Vesicles, Quatsomes, as Scaffolds and Their Use as Probes for Bioimaging. *Small* **2018**, *14* (16), 1703851.

(5) Phillips, J. C.; Braun, R.; Wang, W.; Gumbart, J.; Tajkhorshid, E.; Villa, E.; Chipot, C.; Skeel, R. D.; Kalé, L.; Schulten, K. Scalable Molecular Dynamics with NAMD. *J. Comput. Chem.* **2005**, *26* (16), 1781–1802.

(6) Gullapalli, R. R.; Demirel, M. C.; Butler, P. J. Molecular Dynamics Simulations of DiI-C18(3) in a DPPC Lipid Bilayer. *Phys. Chem. Chem. Phys.* **2008**, *10* (24), 3548–3560.

(7) Cabrera, I.; Elizondo, E.; Esteban, O.; Corchero, J. L.; Melgarejo, M.; Pulido, D.; Córdoba, A.; Moreno, E.; Unzueta, U.; Vazquez, E.; et al. Multifunctional Nanovesicle-Bioactive Conjugates Prepared by a One-Step Scalable Method Using CO2-Expanded Solvents. *Nano Lett.* **2013**, *13* (8), 3766–3774.

(8) Debnath, P.; Chakraborty, S.; Deb, S.; Nath, J.; Bhattacharjee, D.; Hussain, S. A. Reversible Transition between Excimer and J-Aggregate of Indocarbocyanine Dye in Langmuir–Blodgett (LB) Films. *J. Phys. Chem. C* **2015**, *119* (17), 9429–9441.

(9) Mustroph, H.; Reiner, K.; Mistol, J.; Ernst, S.; Keil, D.; Hennig, L. Relationship between the Molecular Structure of Cyanine Dyes and the Vibrational Fine Structure of Their Electronic Absorption Spectra. *ChemPhysChem* **2009**, *10* (5), 835–840.

(10) Terdale, S.; Tantray, A. Spectroscopic Study of the Dimerization of Rhodamine 6G in Water and Different Organic Solvents. *J. Mol. Liq.* **2017**, *225*, 662–671.

(11) Ferreira, L. F. V.; Oliveira, A. S.; Wilkinson, F.; Worrall, D. Photophysics of Cyanine Dyes on Surfaces. A New Emission from Aggregates of 2,2′-Cyanines Adsorbed onto Microcrystalline Cellulose. *J. Chem. Soc. Faraday Trans.* **1996**, *92* (7), 1217–1225.

(12) v. Berlepsch, H.; Böttcher, C. H-Aggregates of an Indocyanine Cy5 Dye: Transition from Strong to Weak Molecular Coupling. *J. Phys. Chem. B* **2015**, *119* (35), 11900–11909.

(13) Patlolla, P. R.; Das Mahapatra, A.; Mallajosyula, S. S.; Datta, B. Template-Free H-Dimer and H-

Aggregate Formation by Dimeric Carbocyanine Dyes. *New J. Chem.* **2018**, *42* (9), 6727–6734.

(14) Kurhuzenkau, S. Dyes and Nanoparticles for Bioimaging: Linear Photophysical and Nonlinear Optical Study, University of Parma, 2017.

(15) Morla-Folch, J.; Vargas-Nadal, G.; Zhao, T.; Sissa, C.; Ardizzone, A.; Kurhuzenkau, S.; Köber, M.; Uddin, M.; Painelli, A.; Veciana, J.; et al. Dye-Loaded Quatsomes Exhibiting FRET as Nanoprobes for Bioimaging. *ACS Appl. Mater. Interfaces* **2020**, *12* (18), 20253–20262.

(16) Anzola, M.; Painelli, A. Aggregates of Polar Dyes: Beyond the Exciton Model. *Phys. Chem. Chem. Phys.* **2021**, No. 23, 8282–8291.

(17) Andreas, R.; S., K. A. Fluorescent Polymer Nanoparticles Based on Dyes: Seeking Brighter Tools for Bioimaging. *Small* **2016**, *12* (15), 1968–1992.

(18) Wu, Y.; Lopez, G. P.; Sklar, L. A.; Buranda, T. Spectroscopic Characterization of Streptavidin Functionalized Quantum Dots. *Anal. Biochem.* **2007**, *364* (2), 193–203.

(19) Bonacchi, S.; Genovese, D.; Juris, R.; Montalti, M.; Prodi, L.; Rampazzo, E.; Zaccheroni, N. Luminescent Silica Nanoparticles: Extending the Frontiers of Brightness. *Angew. Chemie Int. Ed.* **2011**, *50* (18), 4056–4066.

(20) Melnychuk, N.; Egloff, S.; Runser, A.; Reisch, A.; Klymchenko, A. S. Light-Harvesting Nanoparticle Probes for FRET-Based Detection of Oligonucleotides with Single-Molecule Sensitivity. *Angew. Chemie Int. Ed.* **2020**, *59*, 6811–6818.

(21) Li, K.; Qin, W.; Ding, D.; Tomczak, N.; Geng, J.; Liu, R.; Liu, J.; Zhang, X.; Liu, H.; Liu, B.; et al. Photostable Fluorescent Organic Dots with Aggregation-Induced Emission (AIE Dots) for Noninvasive Long-Term Cell Tracing. *Sci. Rep.* **2013**, *3* (1), 1150.

(22) Rampazzo, E.; Boschi, F.; Bonacchi, S.; Juris, R.; Montalti, M.; Zaccheroni, N.; Prodi, L.; Calderan, L.; Rossi, B.; Becchi, S.; et al. Multicolor Core/Shell Silica Nanoparticles for in Vivo and Ex Vivo Imaging. *Nanoscale* **2012**, *4* (3), 824–830.

(23) Larson, D. R.; Ow, H.; Vishwasrao, H. D.; Heikal, A. A.; Wiesner, U.; Webb, W. W. Silica Nanoparticle Architecture Determines Radiative Properties of Encapsulated Fluorophores. *Chem. Mater.* **2008**, *20* (8), 2677–2684.

(24) Ow, H.; Larson, D. R.; Srivastava, M.; Baird, B. A.; Webb, W. W.; Wiesner, U. Bright and Stable Core−Shell Fluorescent Silica Nanoparticles. *Nano Lett.* **2005**, *5* (1), 113–117.

(25) Palantavida, S.; Tang, R.; Sudlow, G. P.; Akers, W. J.; Achilefu, S.; Sokolov, I. Ultrabright NIR Fluorescent Mesoporous Silica Nanoparticles. *J. Mater. Chem. B* **2014**, *2* (20), 3107–3114.

(26) Rampazzo, E.; Bonacchi, S.; Juris, R.; Montalti, M.; Genovese, D.; Zaccheroni, N.; Prodi, L.; Rambaldi, D. C.; Zattoni, A.; Reschiglian, P. Energy Transfer from Silica Core−Surfactant Shell Nanoparticles to Hosted Molecular Fluorophores. *J. Phys. Chem. B* **2010**, *114* (45), 14605–14613.

(27) Mastrodonato, C.; Pagano, P.; Daniel, J.; Vaultier, M.; Blanchard-Desce, M. Molecular-Based Fluorescent Nanoparticles Built from Dedicated Dipolar Thienothiophene Dyes as Ultra-Bright Green to NIR Nanoemitters. *Molecules* **2016**, *21* (9), 1227.

(28) Daniel, J.; Godin, A. G.; Palayret, M.; Lounis, B.; Cognet, L.; Blanchard-Desce, M. Innovative Molecular-Based Fluorescent Nanoparticles for Multicolor Single Particle Tracking in Cells. *J. Phys. D. Appl. Phys.* **2016**, *49* (8), 84002.

(29) Sourdon, A.; Gary-Bobo, M.; Maynadier, M.; Garcia, M.; Majoral, J.-P.; Caminade, A.-M.; Mongin, O.; Blanchard-Desce, M. Dendrimeric Nanoparticles for Two-Photon Photodynamic Therapy and Imaging: Synthesis, Photophysical Properties, Innocuousness in Daylight and Cytotoxicity under Two-Photon Irradiation in the NIR. *Chem. – A Eur. J.* **2019**, *25* (14), 3637–3649.

(30) Mongin, O.; Krishna, T. R.; Werts, M. H. V; Caminade, A.-M.; Majoral, J.-P.; Blanchard-Desce, M. A Modular Approach to Two-Photon Absorbing Organic Nanodots: Brilliant Dendrimers as an Alternative to Semiconductor Quantum Dots? *Chem. Commun.* **2006**, No. 8, 915–917.

(31) Reisch, A.; Didier, P.; Richert, L.; Oncul, S.; Arntz, Y.; Mély, Y.; Klymchenko, A. S. Collective Fluorescence Switching of Counterion-Assembled Dyes in Polymer Nanoparticles. *Nat. Commun.* **2014**, *5*, 4089.

(32) Melnychuk, N.; Klymchenko, A. S. DNA-Functionalized Dye-Loaded Polymeric Nanoparticles: Ultrabright FRET Platform for Amplified Detection of Nucleic Acids. *J. Am. Chem. Soc.* **2018**, *140* (34), 10856–10865.

(33) Wagh, A.; Qian, S. Y.; Law, B. Development of Biocompatible Polymeric Nanoparticles for in Vivo NIR and FRET Imaging. *Bioconjug. Chem.* **2012**, *23* (5), 981–992.

(34) Anil, W.; Faidat, J.; Sanku, M.; Steven, Q.; Estelle, L.; Benedict, L. Polymeric Nanoparticles with Sequential and Multiple FRET Cascade Mechanisms for Multicolor and Multiplexed Imaging. *Small* **2013**, *9* (12), 2129–2139.

(35) Behnke, T.; Würth, C.; Laux, E.-M.; Hoffmann, K.; Resch-Genger, U. Simple Strategies towards Bright Polymer Particles via One-Step Staining Procedures. *Dye. Pigment.* **2012**, *94* (2), 247–257.

(36) Trofymchuk, K.; Reisch, A.; Shulov, I.; Mély, Y.; Klymchenko, A. S. Tuning the Color and Photostability of Perylene Diimides inside Polymer Nanoparticles: Towards Biodegradable Substitutes of Quantum Dots. *Nanoscale* **2014**, *6* (21), 12934–12942.

(37) Kilin, V. N.; Anton, H.; Anton, N.; Steed, E.; Vermot, J.; Vandamme, T. F.; Mely, Y.; Klymchenko, A. S. Counterion-Enhanced Cyanine Dye Loading into Lipid Nano-Droplets for Single-Particle Tracking in Zebrafish. *Biomaterials* **2014**, *35* (18), 4950–4957.

(38) Wang, X.; Anton, N.; Ashokkumar, P.; Anton, H.; Fam, T. K.; Vandamme, T.; Klymchenko, A. S.; Collot, M. Optimizing the Fluorescence Properties of Nanoemulsions for Single Particle Tracking in Live Cells. *ACS Appl. Mater. Interfaces* **2019**, *11* (14), 13079–13090.
